# The impact of shape and attachment position of biologging devices in Northern Bald Ibises

**DOI:** 10.1186/s40317-023-00322-5

**Published:** 2023-03-09

**Authors:** Ortal Mizrahy-Rewald, Natalie Winkler, Frederik Amann, Katharina Neugebauer, Bernhard Voelkl, Herwig A. Grogger, Thomas Ruf, Johannes Fritz

**Affiliations:** 1 Department of Interdisciplinary Life Sciences, University of Veterinary Medicine, Savoyenstrasse 1a, 1160 Vienna, Austria; 2 Waldrappteam Conservation and Research, Schulgasse 28, 6162 Mutters, Austria; 3 Animal Welfare Division, Vetsuisse Faculty, University of Bern, Laenggassstrasse 120, 3012 Bern, Switzerland; 4 Vienna Scientific Instruments, Heiligenkreuzer Strasse 466, 2534 Alland, Austria; 5 Engineering Department, University of Applied Sciences Joanneum, Alte Poststrasse 149, 8020 Graz, Austria; 6 Department of Behavioral and Cognitive Biology, University of Vienna, Djerassiplatz 1, 1030 Vienna, Austria

**Keywords:** Aerodynamic, Biologging, Heart rate, VeDBA, Wind tunnel, Harness, Flight performance

## Abstract

**Background:**

The impact of biologging devices on the aerodynamics or hydrodynamics of animals is still poorly understood. This stands in marked contrast to the ever more extensive use of such technologies in wild-living animals. Recently, increasing concerns have been raised about the impairing effects of these devices on the animals concerned. In the early days of biotelemetry, attention was focused solely on reducing weight, but now aerodynamic effects are also increasingly being considered. To investigate these effects, we trained Northern Bald Ibises to fly in a wind tunnel in which we measured heart rate and dynamic body acceleration (VeDBA) as proxies for energy expenditure in relation to different logger shapes and wind flow directions.

**Results:**

Our data provide evidence that the position of biologging devices significantly influence the flight distances, and the shape of biologging devices has a considerable effect on heart rate and VeDBA, both of which have been used as proxies for energy expenditure. Unfavorable shape and positioning go beyond merely affecting the effort required during flapping flight. The energetically probably more important effect is that the devices impair the bird’s ability to glide or soar and thus force them to perform the energetically much more demanding flapping flight more frequently. This effect was more pronounced in rising air than in horizontal airflow. A complementary study with wild Northern Bald Ibises during spring migration provides evidence that the position of the devices on the bird’s back affects the length of the flight stages. Birds carrying the devices on the upper back, fixed by wing-loop harnesses, had significantly shorter flight stages compared to birds with a more caudally positioned device, fixed by leg-loop harnesses.

**Conclusion:**

The attachment of biologging devices on birds affects their performance and behavior and thus may influence their fitness and mortality. Our results show that detrimental effects can be reduced with relatively little effort, in particular through a strictly aerodynamic design of the housing and increased consideration of aerodynamics when attaching the device to the body. In birds, the attachment of biologging devices via leg loops to the lower back is clearly preferable to the common attachment via wing loops on the upper back, even if this affects the efficiency of the solar panels. Nevertheless, the importance of drag reduction may vary between systems, as the benefits of having a biologging devices close to the center of gravity may outweigh the increase in drag that this involves. Overall, more research is required in this field. This is both in the interest of animal welfare and of avoiding biasing the quality of the collected data.

**Supplementary Information:**

The online version contains supplementary material available at 10.1186/s40317-023-00322-5.

## Background

The study of large-scale animal movement is fundamental to our understanding of animal ecology, behavior, and conservation [1–3]. Biologging devices attached to wild animals to measure their geolocation, acceleration, heart rate, and other biometric as well as environmental parameters can provide us with increasingly extensive and precise parameters [4–6]. The last few decades have been marked by a technological revolution in this field of research. Devices have become continuously smaller and lighter, enabling tracking smaller animals and equipping the devices with increasingly diverse sensor and processing technology [5, 6]. Placement varies widely from subcutaneously or intraperitoneally implanted devices to glued devices, collars, and harnesses that fasten the devices to the animal [6–9].

The increasing application of biologging technology has raised awareness for ethical concerns arose, and impairing impacts of biologging have been documented in various studies. The effects range from breeding success, energy expenditure, behavior, functionality of the sensory systems to the survival rate of the animals [10–18]. Apart from the ethical considerations, the data collected from the tracking devices can lead to biased results and questionable conclusions because they disrupt the animal [6, 19]. These impacts have been especially studied in flying animals because biologging is crucial to study their movement. The additional mass of the device and the higher drag it creates can affect the performance and reproductive success of the animal [11, 13, 20, 21].

Nevertheless, studies utilizing biologging technologies continue to be published that only apply the rule that the device must weigh less than 3–5% of the total body mass, even though this rule has been repeatedly criticized for not being evidence based [11, 12, 17, 22, 23]. Moreover, solely considering the weight of the devices ignores other potentially detrimental parameters, such as the size, shape, and position on the animal’s body. Hence, an increasing number of researchers are advocating that studies using biologging devices should regard, evaluate, and publish—apart from weight—further potentially detrimental parameters [7, 15, 23].

When using a harness to attach a device to a bird’s body, the most common placement methods are on the upper back (wing-loop harness) and the lower back (leg-loop harness). Pennycuick and colleagues [24] investigated in a wind tunnel how the attachment of various devices with wing-loop harnesses increased the drag coefficient of Rose-colored Starlings (*Sturnus roseus*) due to the added frontal area and the separation of the boundary layer over the caudal upper body of the birds. They found that devices increased the drag by up to nearly 50%, and adding a sloping antenna increased it to nearly twice the base value. They concluded that the increase in power requirements reduces the range of migrating birds and the reserves remaining on arrival. This effect increased with migration distance. Leg-loop harnesses are assumed to have less impact on drag coefficient during flight because they are less exposed to the airstream during the flight [10, 18, 24]. Nonetheless, in birds, devices continue to frequently be attached at the front position via wing-loop harnesses. This is because most devices are equipped with solar panels, which are better supplied with sunlight in this exposed position [18, 25]. This position, however, has disadvantages that go beyond the obvious aerodynamic issues. In Northern Bald Ibises, there is a correlative relationship between biologging devices on this upper-back position and a progressive opacity of the eyes’ cornea up to, and including, blindness [18]. When the devices are removed from this position, these symptoms can disappear again provided the eyeball has not already been irreversibly damaged [18].

Apart from a device’s position, its size and shape have a significant impact on the drag coefficient. Here, a streamlining shape, proper dimensions, and a reduced frontal area reduce drag [26]. In an experimental wind tunnel setup, Obrecht and colleagues [26] found that adding a rounded fairing to the front end and a pointed fairing behind helped reduce the drag of the transmitter by about one-third compared to a rectangular box. In a study by Kay et al. [23] a computational fluid dynamic was used to simulate the water flow along a seal’s body in order to investigate the effects of differently shaped devices attached to the body. They found that a conventionally shaped tag induces up to 22% more drag compared to a streamlined tag, whereby the conventional tag’s size would have to be reduced by 50% to match the drag of the streamlined version. Moreover, the effect on the induced drag by changing the attachment position on the seal's body differed much less in streamlined versus conventional tags.

In this study, we explored the effect of a conventional rectangular housing (cube shaped) and an aerodynamically shaped housing (drop shaped) with trained Northern Bald Ibises (*Geronticus eremita*) flying in a wind tunnel [27]. We measured heart rate and overall dynamic body acceleration (VeDBA) as proxies for energy expenditure [28–30], as well as the flapping and gliding proportion. The movable outlet nozzle of the wind tunnel [27] enabled testing the birds in both a horizontal airflow and an ascending airflow (which favors gliding flight). In addition to this experimental setup, a reintroduction project provided the framework for an in situ study. Here, we compared the flight performance of Northern Bald Ibises during spring migration with differently attached biologging devices (wing loop vs. leg loop).

## Methods

### Wind tunnel experiment

**Fig. 1:**
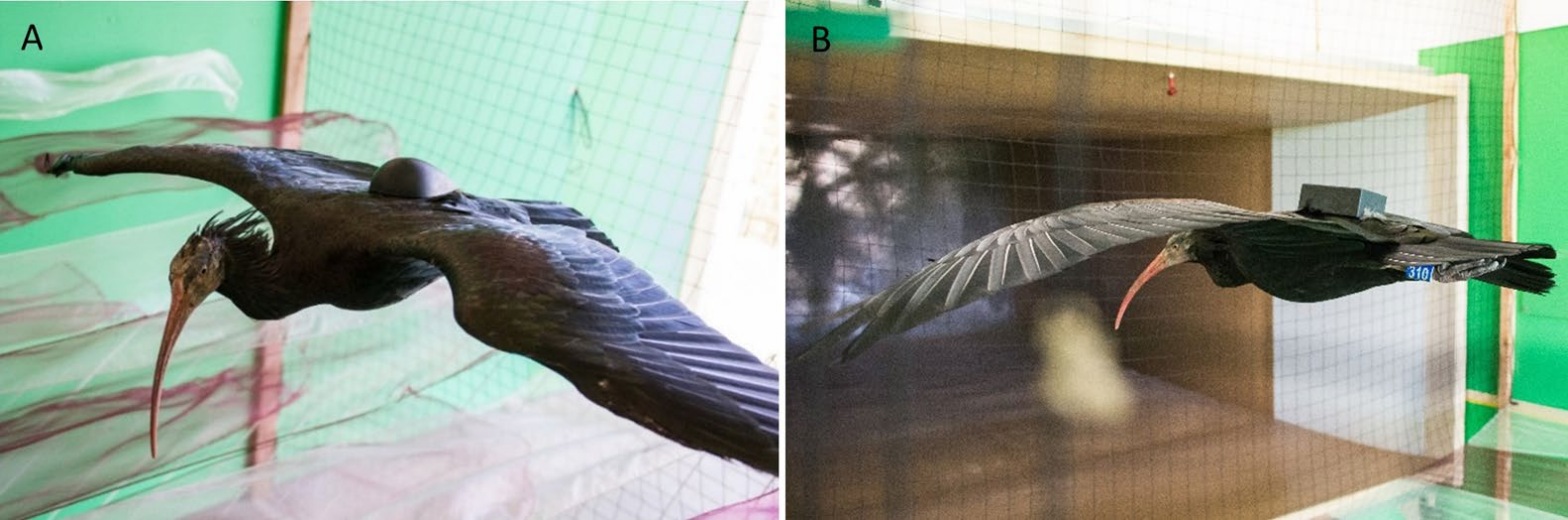
**A** 3D printed drop-shaped (aerodynamic) cover mounted on a flying Northern Bald Ibis. **B** 3D printed cube-shaped (non-aerodynamic) cover mounted on a flying Northern Bald Ibis. Photos were taken by Waldrappteam Conservation & Research in the Seekirchen wind tunnel (AT)

The experimental study was done in a blower-type wind tunnel, set up in a private research center of *Waldrappteam Conservation & Research*, located in Seekirchen am Wallersee, Salzburg, Austria (Fig. 1). The test section of the wind tunnel measures 2.5 m × 1.5 m and the maximum achievable flow speed is approximately 16 ms^−1^, which fits well with the flight speed of this species [27]. The outlet nozzle has flexible walls at the bottom and on the top. They can be adjusted in parallel to produce downdrafts (max. − 5°) or updrafts (max. + 7°) for gliding and climbing flights [27].

In spring 2019, two experienced members of *Waldrappteam Conservation & Research* received four Northern Bald Ibis chicks from Zurich Zoo for hand raising, following a detailed raising protocol [31]. From July 2019, the fledged, human-imprinted juveniles were gradually trained by their two foster parents to fly in the wind tunnel, with each animal trained individually. Training methods comprised socio-positive interactions with the foster parents using gradual habituation and operant conditioning [27, 32]. Since the open circuit blower-type wind tunnel is subject to changing meteorological conditions, training had to be interrupted from late autumn 2019 until spring 2020 due to low temperature and high humidity.

The data collection took place in late summer 2020. Two male birds (No. 310 and 311) that flew particularly reliably in the test room were chosen for the experiments. Each bird flew in four conditions that combined two different housing shapes—aerodynamic (drop shape) and non-aerodynamic (cube shape) (dimension: 12 × 5 × 3 cm, Fig. 1, Additional file 1: Fig. S1)—with two different angles of the outlet nozzle: (horizontal flow) + 2° and (updraft flow) + 6°. Each test session consisted of two minimum 5 min consecutive flights with approximately 5 min break in between. A maximum of two sessions per day were performed by the birds. Prior to the data collection, a leg-loop harness was strapped to the birds to fix a plastic plate (80 × 35 mm) on their lower back (5.8 g in total for the harness and the plastic plate; Fig. 1 and Additional file 1: Fig. S1). Before each flight session, the trainers (foster parents) attached a data logger, three adhesive mini electrodes (see below), and one of the two housings (i.e., drop or cube shape) on the plastic plate (Additional file 1: Fig. S1). Thus, the location of the data logger was fixed always at the same place, at the middle of the housing just above the plastic plate.

After a flight session, only the loggers, electrodes, and housing were removed, while the harness and plastic plate stayed fixed to the birds and were removed only at the end on the data collection. 

### Heart rate and acceleration measurement

Heart rate was recorded at a measurement frequency of 1600 Hz using the external ECG logger Neurologger 2A (Additional file 1: Fig. S1) with a 1 GB memory (Evolocus LLC, NY, USA) [33–35]. To the ECG loggers, we connected three lead wires with adhesive mini electrodes (NeoLead^®^, ©Connect Medizintechnik GmbH, Mistelbach, Austria) soldered to the ends (Additional file 1: Fig. S1). The logger recorded the electric potential difference between two electrodes at a range of − 6 to + 6 mV, with the third electrode serving as a reference electrode. The first two electrodes were attached to the skin on each side of the lower abdomen, the third (reference electrode) to the middle of the back.

The Neurologger 2A was equipped with an on-board accelerometer (LIS302DLH; STMicroelectronics, Geneva, Switzerland) that recorded three axial accelerations between g-forces of − 8 and + 8 g at 1600 Hz. The body of the logger was fixed on the leg-loop harness. The heart rate and accelerometer logger unit, including ECG wires, electrodes, battery, the harness, and the plastic plate, weighed 15.94 ± 0.1 g. The logger was covered by one of the two 3D printed housings: drop shaped (18.3 ± 0.1 g) or cube shaped (22.4 ± 0.1 g) (Fig. 1 and Additional file 1: Fig. S1). As the two covers did not have the same weight, we attached a small metal piece inside the drop-shaped cover to match the weight of the cube-shaped cover. Hence, the overall weight that the birds carried during the data collection reached a maximum of 38.34 ± 0.1 g, equivalent to almost 3% of the birds’ mean body mass (1300 g) and thus below the commonly recommended value of 3%.

### Heart rate and accelerometer analyses

We processed the ECG and acceleration data using R v. 4.05 [36]. The analysis process is described in detail in Mizrahy-Rewald et al. (2022) and [37]. In brief, we used a custom QRS complex detection algorithm in R to calculate the heart rate in beats per minute (bpm) from the raw ECG data (in mV). The calculation took into consideration the respective activity of the bird, based on the heave axis from the acceleration data. Each data point was ranked based on its neighbor counts and only data points with the highest ranks were kept (i.e., points within a dense group). We then visualized the data and deleted noticeable outliners. In this study, we statistically analyzed the processed heart rate data in bpm with a final frequency of 5 Hz.

Using the tri-axial acceleration data, we calculated the vector of the dynamic body acceleration (VeDBA, m*g*). We first calculated the static acceleration by smoothing each acceleration axis over a window size of 1 s using a running mean. Then we calculated the dynamic acceleration by subtracting the static acceleration from the raw acceleration values. We summed the squares of all three axes’ dynamic body acceleration and square root it to obtain the VeDBA [38–40]. Finally, we downscaled the VeDBA data by sub-sampling it to 1 Hz to meet the same timesteps of the processed heart rate data.

Flapping and gliding were identified based on the accelerometer data and using a custom algorithm in R (described in Mizrahy-Rewald et al. 2022). A threshold of the absolute value of the heave axis (i.e., 300 mg) was identified to distinguish between flapping and gliding. Gliding was defined when the absolute value was below the threshold for a minimum of one wingbeat (0.2 s). After the above identification, the dataset of flapping and gliding was merged with the heart rate and VeDBA data based on the timestamps of the latter data. Thus, the final datasets have a temporal resolution of 1 Hz.

### Statistics

All statistical analyses were done using R v. 4.05 [36].

The effect of different logger shapes and wind angles on VeDBA, heart rate, and VeDBA during flapping was analyzed using a Generalized Additive Mixed Model (GAMM) with the function ‘bam()’ within the package ‘mgcv’ [41] (formulas 1, 2, 4; Table 1). GAMM provides a general framework by enabling linear and nonlinear functions of each of the variables while maintaining the additive assumption. In addition, it offers flexibility through smooth functions that can be applied to each explanatory variable [42]. This experiment was set up as a crossed design with repeated measures, meaning that the two birds flew in all four conditions but not necessarily with the same condition at the same day (Table 2). The response variables (heart rate and OBDA) contained various degrees of autocorrelation, which was corrected by including autoregressive order 1 (AR1) error models in GAMM functions. This successfully reduced the autocorrelation to non-significant levels, which we confirmed by comparing the autocorrelation function of model residuals (ACF) before and after the correction.

**Table 1:** Models for explaining energy expenditure based on heart rate and VeDBA, gliding proportion and flight performance in Northern Bald Ibises

Formula 1 GAMM: HR ~ Logger shape × Wind angle + flight number + s(Bird, bs = “re”) + s(Date, bs = “re”, by = Logger shape) + s(Date, bs = “re”, by = Wind angle)
Formula 2 GAMM: VeDBA ~ Logger shape × Wind angle + flight number + s(Bird, bs = “re”) + s(Date, bs = “re”, by = Logger shape) + s(Date, bs = “re”, by = Wind angle)
Formula 3 LM: Gliding frequency ~ Logger shape × Wind angle + Bird
Formula 4 GAMM: VeDBA during flapping ~ Logger shape × Wind angle + flight number + s(Bird, bs = “re”) + s(Date, bs = “re”, by = Logger shape) + s(Date, bs = “re”, by = Wind angle)
Formula 5 LMER: log10(Distance) ~ Logger position × Activity + (1 |Bird)

GAMMs to explain heart rate (HR) and overall dynamic body acceleration (VeDBA) under different flying conditions (formulas 1, 2, 4). Linear regression to explains the gliding proportion under different flying conditions (formula 3). Linear mixed model to explain the flight performance with different harness attachments (formula 5). bs = “re”: basis specification for random effects

**Table 2:** Overview of the wind tunnel experiments with all four combinations of housing shape (drop shape and cube shape) and wind angle (horizontal flow at + 2° and significant updraft at + 6°)

Condition (housing, wind angle)	Number of flights; Bird 310 (*n*)	Number of flights; Bird 311 (*n*)	Total recorded data (seconds)	Mean heart rate (bpm)	Mean VeDBA (m*g*)
cube shape, wind + 2°	6	8	4469	461	793
drop shape, wind + 2°	8	6	4496	443	672
cube shape, wind + 6°	6	6	3829	414	492
drop shape, wind + 6°	6	6	4092	335	331

We included a categorical variable for ‘flight number’ (two flights in each session) and an interaction term between ‘logger shape’ and ‘wind angle.’ The gam function computes a model with a fixed effect for each level of the category. We used a smooth term for the categorical predictor (‘Bird’) with a basis specification for random effects s(Bird, bs = “re”) to allow for differences in the mean level of heart rates and VeDBA between individuals. Although we collected data from only two birds, which is below the advised minimum number of groups for a multilevel model, using two groups adds little over classical models but does no worse than no-pooling regression [43]. The flying conditions (logger shape and wind angle) varied between days. We therefore expect ‘Days’ (*n* = 14) to have different variances for each logger shape and wind angle and thus incorporated the random effect of ‘Days,’ allowing for different smoothness parameters for each logger shape and wind angle [e.g. s(Date, by = Logger shape, bs = “re”)]. The default selection score in GAMM [i.e., fast restricted maximum likelihood (fREML)] was applied. We fitted the ‘heart rate model’ and ‘VeDBA model’ using the scaled-*t* (Student’s *t*) family distribution, given that the pattern of the residuals, as inspected by normal quantile–quantile and histogram plots, resembled that of a normal distribution with heavier tails. The ‘VeDBA during flapping model,’ was fitted using the Gaussian family distribution. In addition, the ‘identity’ link function was specified for all models. The function ‘gam.check’ in the ‘mgcv’ package was used for model diagnostics, and the model-checks graphs were plotted using the function ‘getViz’ in the ‘mgcViz’ package [44], which is an extension of the ‘mgcv’ package (Additional file 1: Fig. S4, S6, and S8). The adjusted coefficient of determination (R^2^adj) was used to measure the goodness-of-fit of the model.

To analyze the effect of the different logger shapes and wind angles on the proportion of gliding, we summarized flapping and gilding frequency for each flying condition. Here, each observation corresponded to one flight session, using ‘groupin by’ function from package ‘dplyr’ [45]. Statistical analysis was performed only on the different gliding proportions between each flying condition, using a multiple linear regression model (formula 3; Table 1). The different logger shapes, wind angles, their interaction, and birds were used as predictor variables.

### Field study during migration

The part of the study with wild-living Northern Bald Ibises took place within a long-term conservation program that aims to re-establish a migratory population in Europe, embedded in the European LIFE program (LIFE + 12-BIO_AT_000143; LIFE20 NAT/AT/000049; *Waldrappteam Conservation & Research*). In late 2021, the population consisted of 199 wild-living individuals, which migrate between breeding sites on the northern foothills of the Alps and the WWF Oasis Laguna di Orbetello in southern Tuscany as the common wintering site [1, 31].

Since 2016, most individuals of the migratory population have been equipped with GPS devices [18] to monitor the birds during their migration journeys and help take efficient measures against illegal bird hunting and electrocution as the main mortality causes [18]. In this data collection, all tags were solar powered (Ornitela, Vilnius, Lithuania) and were positioned on the back of the birds and fixed with a Teflon tube harness, either on the upper back via wing-loop harness or on the lower back via leg-loop harness. The weight of the GPS tracker with the harness was about 25 g, which is 1.9% of the birds’ mean body mass (1300 g) and thus well below the commonly recommended maximum value of 3% [11].

We used GPS data from 37 individuals during spring migration in 2018 and 2019. The birds’ flights led from Tuscany over about 800 km to one of the two breeding areas, either Burghausen in Bavaria (GER) or Kuchl in the province of Salzburg (AUT). Of these 37 birds, 10 individuals carried their device with a wing-loop harness on the upper back and 27 individuals with a leg-loop harness on the lower back.

The data are automatically transferred to the open-source animal movement platform Movebank (movebank.org) as well as to the internal database owned by the enterprise *Waldrappteam Conservation& Research*. All analyses for this study were done using data from the internal database.

### Location and distance traveled analysis

To calculate daily flight distances (Additional file 1: Table S1), we used the open-source Geographic Information System QGIS, Version 3.10.7 ‘A coruña’ [46]. The Northern Bald Ibis usually migrates in several stages, partly with one or more stopovers days in between, during which the birds fly only for small foraging trips [47]. To distinguish between migratory versus stopover days, we set a minimum threshold of 50 km travel distance per day for migration days. We defined the starting date of migration as the day when a bird flew a minimum of 50 km for at least two consecutive days in direction of their breeding site.

### Statistics

A total of 1060 bird-days were used for the statistical analysis (leg loop: 728 days, wing loop: 332 days). On average, 82 GPS fixes per day were collected from birds with wing-loop devices and 52 GPS fixes per day for birds with leg-loop devices (summarized information, see Additional file 1: Table S2). For each bird and day, we calculated the length of the traveled distance as the length of the flight path given by connecting all available GPS fixes. We first examined the frequency distributions of daily flight distances of all birds and days. As the distribution of these distances was strongly skewed, we log-transformed the flight distance data. To analyze the effect of the two different logger positions, we used a mixed effect linear regression model (GLM) fitted with restricted likelihood (REML) (formula 5; Table 1). For the mixed effect model, the package ‘lmerTest’ was used [48]. The response variable of traveled distance (‘Distance’) was predicted using the fixed factors ‘harness position’ and ‘activity’ (i.e., stopover, or migratory days) and the interaction between them. ‘Bird’ was used as a random factor. The pattern of the residuals, as inspected by normal quantile–quantile plots, resembled that of a normal distribution.

All predicted fitted graphs were created using the ‘visreg’ function from package ‘visreg’ [49]. To visualize the effects of fixed effects in mixed models (i.e., the GAMMSs and GLM: formulas 1, 2, 4, 5), we produced contrast plots (differences between each level of the factor and the reference category) because ‘visreg’ is restricted by incorporating uncertainty about random effects into the predictions from a frequentist perspective. In contrast plots, the confidence interval for the reference categories is zero, while there is no uncertainty about how the expected value of response will change at the same level of the reference category. For the regression model (formula 3), we produced conditional plots. The confidence intervals in all figures are Wald confidence intervals.

### Ethical note

Bird care, keeping, training, and release followed well-established standards in accordance with the legal framework and under the supervision of *Waldrappteam Conservation & Research* experts. All translocation and management measures were implemented in the framework of the European LIFE + reintroduction (LIFE + 12-BIO_AT_000143. National approvals were provided by the provinces of Salzburg (21302-02/239/352-2012) and Carinthia (11-JAG-s/75-2004), as well as by Baden-Württemberg (I1-7.3.3_Waldrapp), Bavaria (55.1- 8646.NAT_03-10-1), and Italy (0027720-09/04/2013).

## Results

### Wind tunnel experiment

#### Logger shape and wind angle

**Fig. 2:**
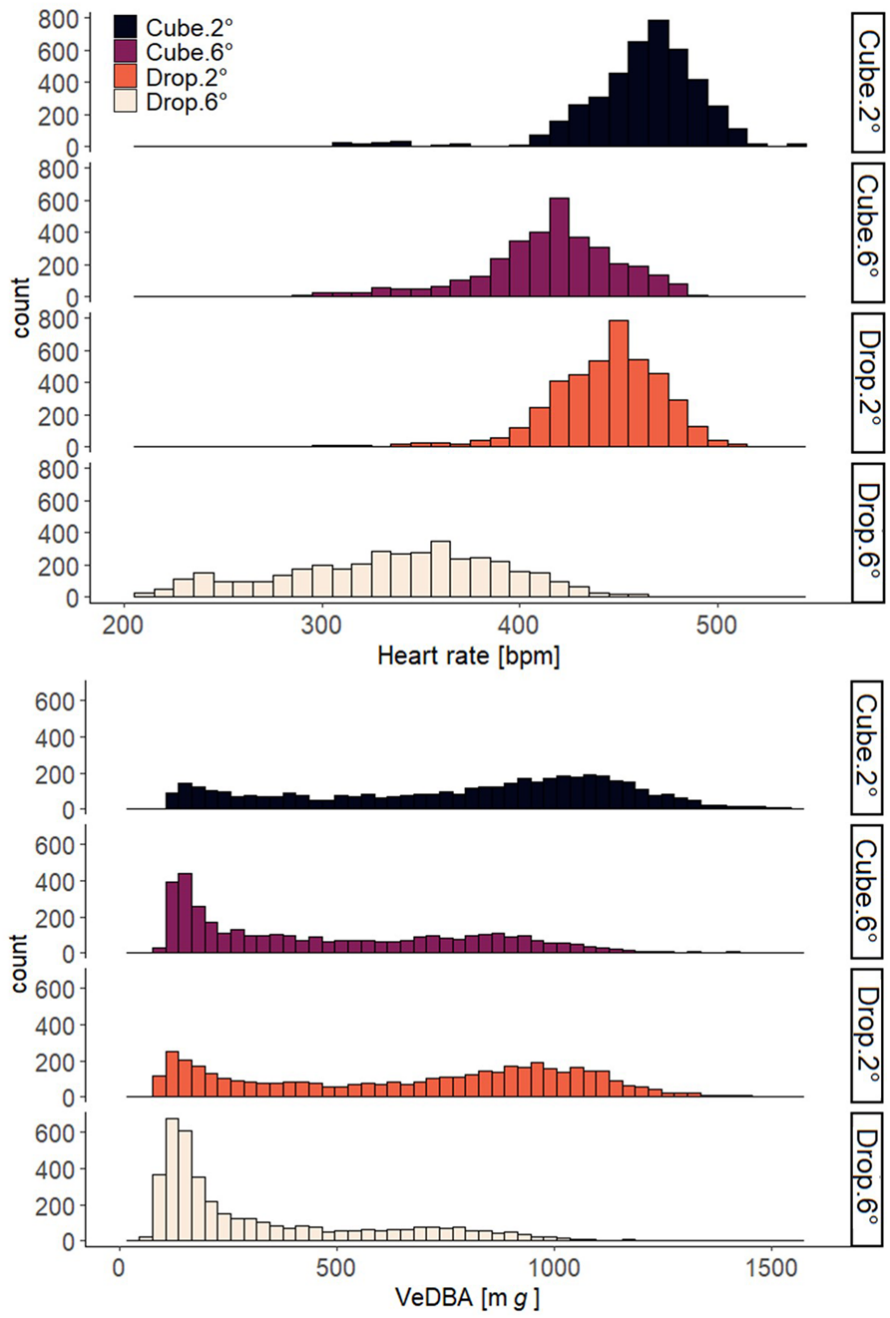
Frequency distribution for Northern Bald Ibises flying in the wind tunnel (*N* = 2 birds) of **(A)** heart rate (bpm) and **(B)** VeDBA (m*g*); different bar colors indicate measurements during flight with different logger shapes (cube and drop) and at different wind angles (+ 2° and + 6°)

The two Northern Bald Ibises flying in a wind tunnel were equipped with either aerodynamic drop-shaped or non-aerodynamic cube-shaped loggers and flew at two wind angles (+ 2° and + 6°). The mean heart rate and VeDBA of the birds ranged from 335 to 461 bpm and 331 to 793 mg, respectively, depending on the logger type and wind angle (Fig. 2, Table 2). The frequency distribution of VeDBA when the birds flew with a drop-shaped logger at a wind angle of + 6° (Drop. + 6°) was left-skewed, suggesting they glided more. For the other flying conditions, we observed a bimodal distribution, suggesting two underlying unimodal distributions of flapping and gliding (Fig. 2B).

**Fig. 3:**
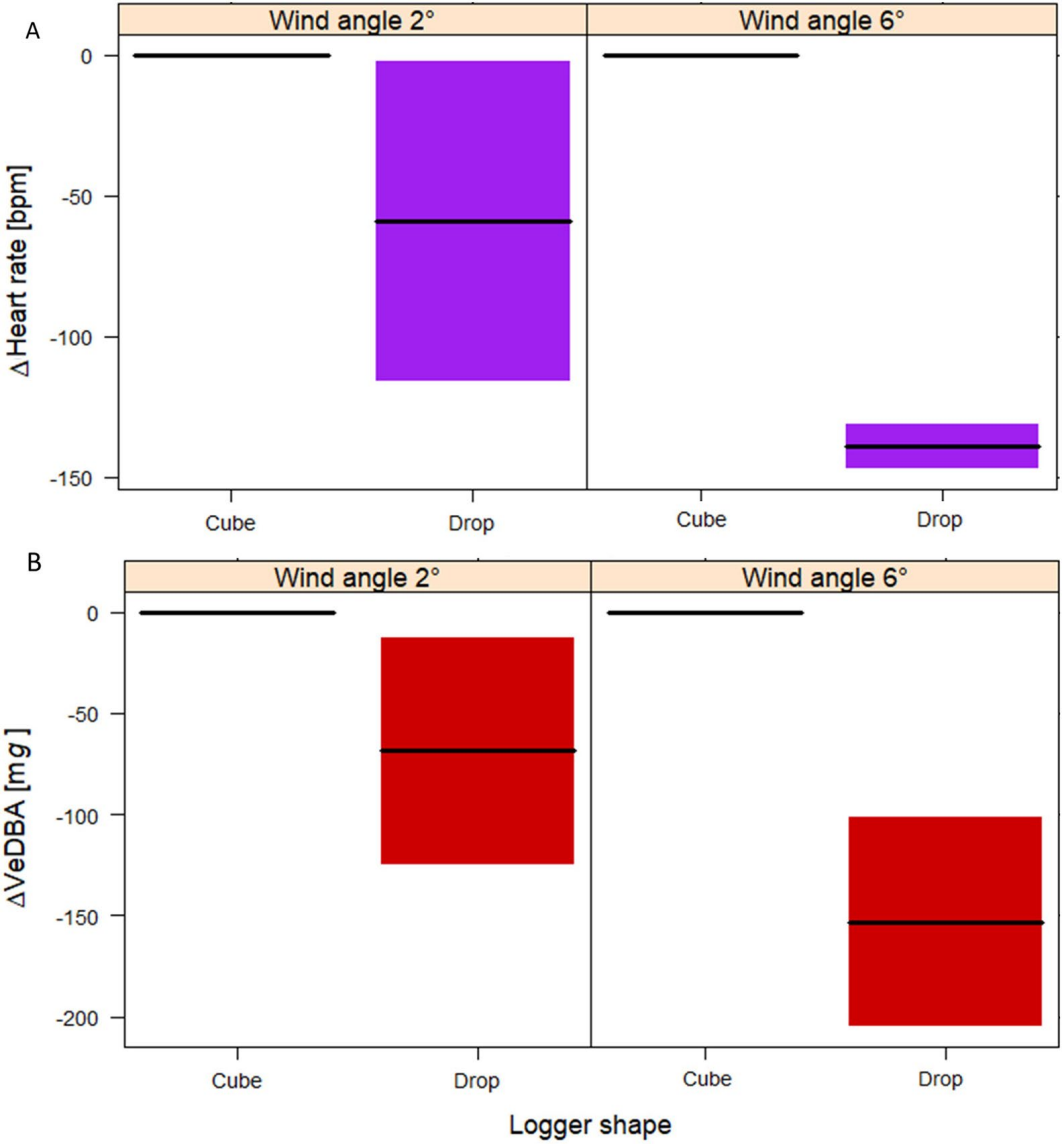
Contrast cross-sectional plots depicting the fixed effect of the GAMMs for **(A)** Δ heart rate (bpm) and **(B)** Δ VeDBA (m*g*), with an interaction between the categorical term ‘wind angle’ and the categorical term ‘logger shape’ (aerodynamic drop and non-aerodynamic cube) on the horizontal axis; wind angle: horizontal flow at + 2° and significant updraft at + 6°; colored areas around the lines: 95% confidence intervals

The fitted GAMM to measure the effect of different logger shapes and wind angles on heart rate had a high explanatory power (*R*^2^_adj_ = 0.72, Table 3). The heart rate did not differ between the two logger shapes when the birds were flying at a wind angle of + 2° (*t* = − 0.77, Fig. 3). At a wind angle of + 6°, however, the difference was significant between the logger shapes. Thus, birds had a significantly lower heart rate when flying with a drop-shaped versus cube-shaped logger (*t* = − 6.464, *p* < 0.001) (Fig. 3, Table 3). Moreover, we measured a slight but significant increase in heart rate between the two flights of the sessions (*t* = 5.96, *p* < 0.001, Additional file 1: Fig. S5). The confidence interval for the reference categories (Cube + 2° and Cube + 6°) is zero because there is no uncertainty about how the expected heart rate value will change at the same level of wind angle.

**Table 3:** Results from the GAMMs multiple regression models testing for the effects of different logger shapes (cube and drop) and wind angles (+ 2° and + 6°) on heart rate and VeDBA during flight

Heart rate				VeDBA			
Linear terms	Estimate	SE	*t*-value	Linear terms	Estimate	SE	*t*-value
Intercept	459.62	23.01	19.96***	Intercept	765.71	32.16	23.81***
Logger Drop	− 13.85	18.21	− 0.77	Logger Drop	− 73.82	23.44	− 3.15**
Wind angle 6°	− 42.08	21.02	− 2.03*	Wind angle 6°	− 283.41	24.31	− 11.67***
Logger Drop:Wind angle 6°	− 79.73	28.78	− 2.77**	Logger Drop:Wind angle 6°	− 84.32	34.17	0.84*
Flight n. 2	6.47	1.09	5.96***	Flight n. 2	12.42	14.77	0.84

Smooth random terms		EDF	*F*-value	Smooth random terms		EDF	*F*-value
s(Bird)		0.99	104,969	s(Bird)		0.91	5.58**
s(Date): LoggerCube		9.30	49,680	s(Date):LoggerCube		0.002	0.00
s(Date): LoggerDrop		9.30	11,828	s(Date):LoggerDrop		1.59	0.17
s(Date):Wind2°		0.00	0	s(Date):Wind2°		1.03	0.18
s(Date):Wind6°		1.16	1278	s(Date):Wind6°		0.001	0.00
*R*^2^_adj_	0.72			*R*^2^_adj_	0.22		
*n*	15,926			*n*	16,880		
fREML	16,901			fREML	14,084		

*edf* effective degrees of freedom of the smooth function terms, *fREML* restricted maximum likelihood; *p < 0.05; **p < 0.01; ***p < 0.001

The change in VeDBA during flight as affected by the different logger shapes and wind angles was fitted with GAMM, which had a low explanatory power (*R*^2^_adj_ = 0.22, Table 3). This is most likely because VeDBA is more strongly affected by the change between flapping and gliding. Nevertheless, logger shape and wind angle had a significant effect (Fig. 3). The VeBDA values were highest when flying with a cube-shaped logger at a wind angle of + 2°. At the same wind angle, flying with a drop-shaped logger significantly reduced VeDBA (*t* = − 3.15, *p* < 0.001). The + 6° wind angle, which allowed more gliding, decreased VeDBA (*t* = − 11.76, *p* < 0.001) even when flying with the non-aerodynamic logger. The lowest VeDBA values were measured when the birds were flying with the aerodynamic logger at a wind angle of + 6° (*t* = 0.84, *p* < 0.05).

### Flapping and gliding

**Fig. 4:**
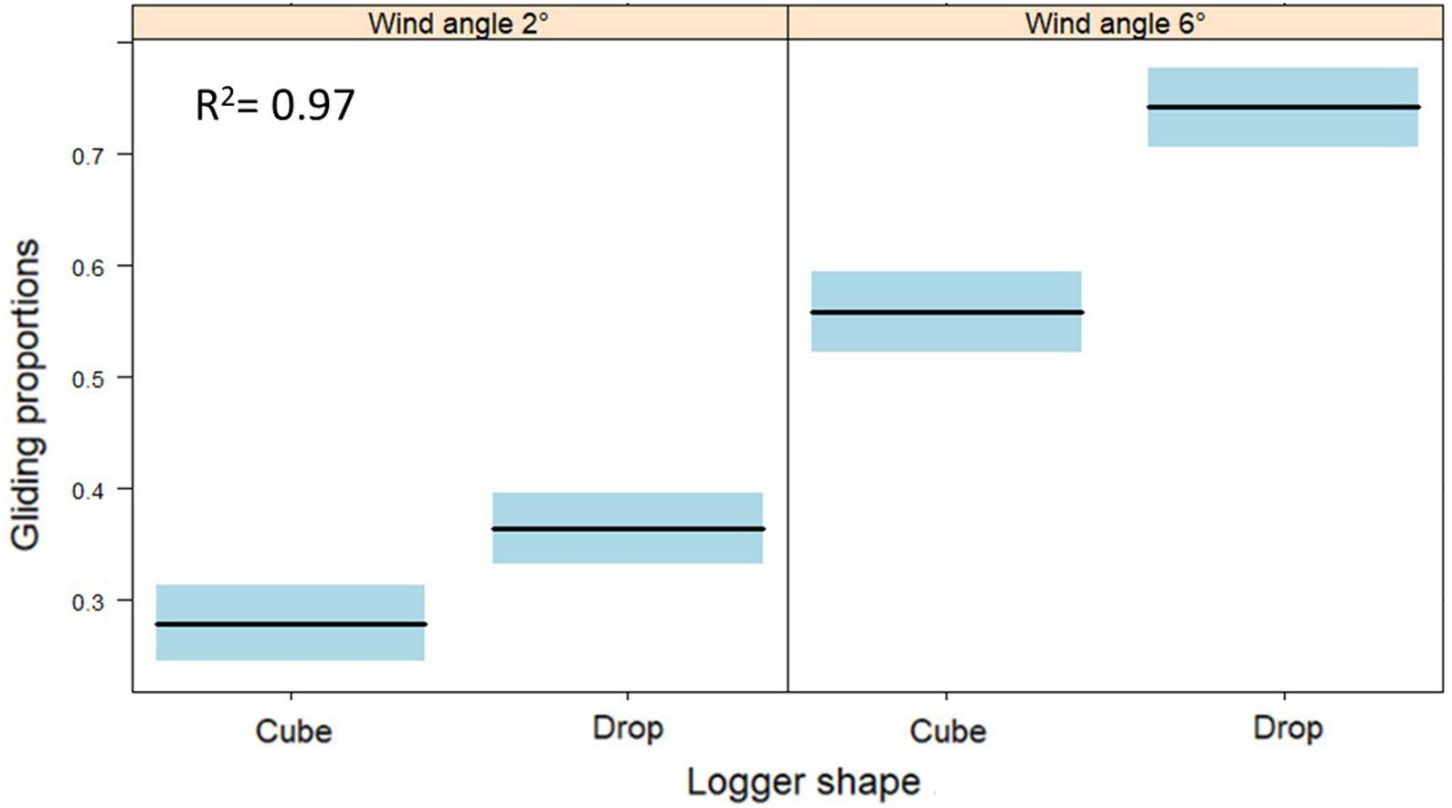
Conditional cross-sectional plots depicting a regression function for gliding proportions that involve categorical explanatory variables, with an interaction between the ‘wind angle,’ and ‘logger shape,’ on the horizontal axis; horizontal flow at + 2° and significant updraft at + 6°; logger shape: cube = non-aerodynamic, drop = aerodynamic; colored areas around the lines: 95% confidence intervals

Multiple linear regression was used to test whether logger shapes and wind angles significantly predicted the gliding proportion during flight. The overall regression was statistically significant (*R*^2^ = 0.97, *F*(4, 21) = 149.9, *p* = 4.042e-15, Table 4). The proportion of gliding was higher when flying at a wind angle of + 6° versus + 2° (*t* = 13.275, *p* < 0.001, Fig. 4). At both wind angles, the gliding proportion differed with logger shape (*t* = 4.180, *p* < 0.001), whereby the drop-shaped form caused a higher gliding proportion. The magnitude of the effect differed between the two wind angles (*t* = 3.315, *p* < 0.01), with a significantly smaller difference at an angle of + 2° compared to + 6°. The highest gliding proportion was measured with drop-shaped loggers at a wind angle of + 6°, the lowest with a cube-shaped logger at + 2°.

**Table 4:** Results of the LM testing for the proportion of gliding at different logger shapes (cube and drop) and different wind angles (+ 2° and + 6°); standard errors (SE); lower and upper confidence intervals (CI); *p < 0.05; **p < 0.01; ***p < 0.001

Terms	Estimate	SE	Lower CI	Upper CI	*t*-value
Intercept	0.280	0.016	0.245	0.312	16.860***
Logger Drop	0.085	0.020	0.043	0.127	4.180***
Wind angle 6°	− 42.08	0.021	0.235	0.322	13.275***
Bird 311	− 0.062	0.015	− 0.093	− 0.032	− 4.200**
Logger Drop × Wind angle 6°	0.099	0.030	0.037	0.160	3.315**

**Fig. 5:**
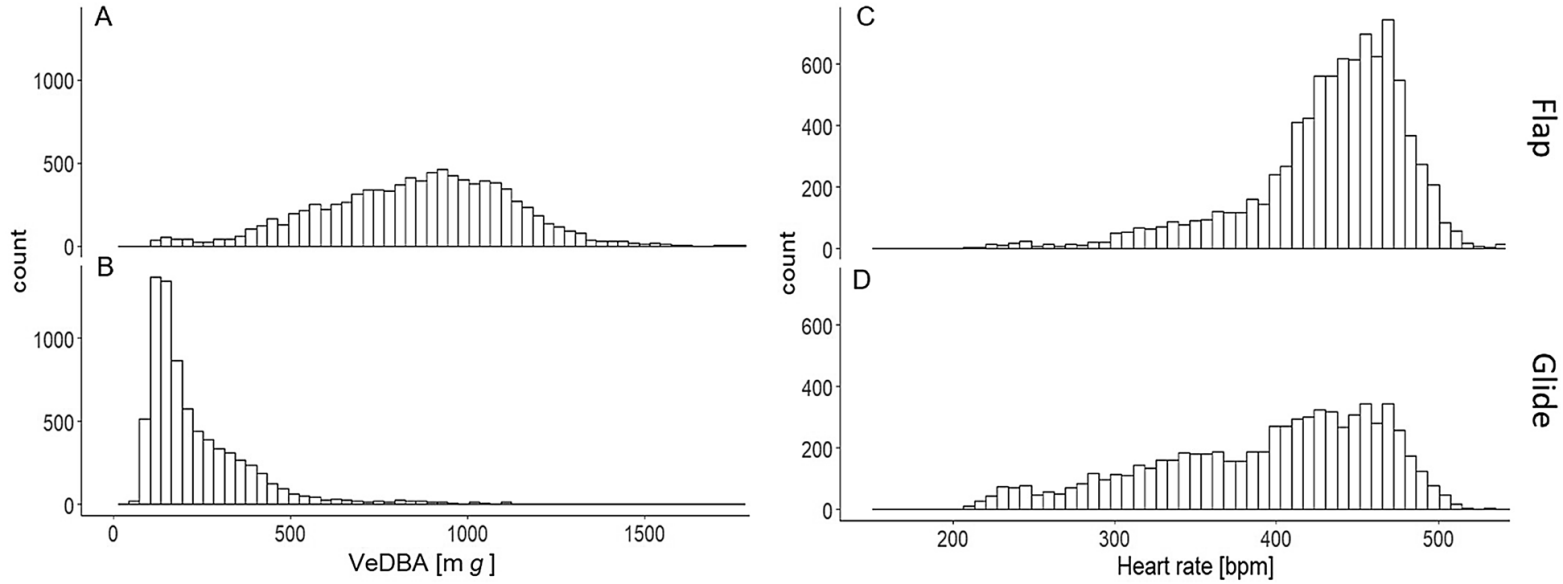
Frequency distribution, for two Northern Bald Ibises flying in the wind tunnel, of (**A**) VeDBA (m*g*) during flapping (**B**) VeDBA (m*g*) during gliding (**C**) heart rate (bpm) during flapping, and (**D**) heart rate (bpm) during gliding

The mean heart rate of the two birds was 433 bpm during flapping and 393 bpm during gliding (Fig. 5). The heart rate frequencies during gliding were distributed over a wide range, indicating a larger variation compared to flapping [35]. The mean VeDBA was 855 mg during flapping and 240 mg during gliding.

**Fig. 6:**
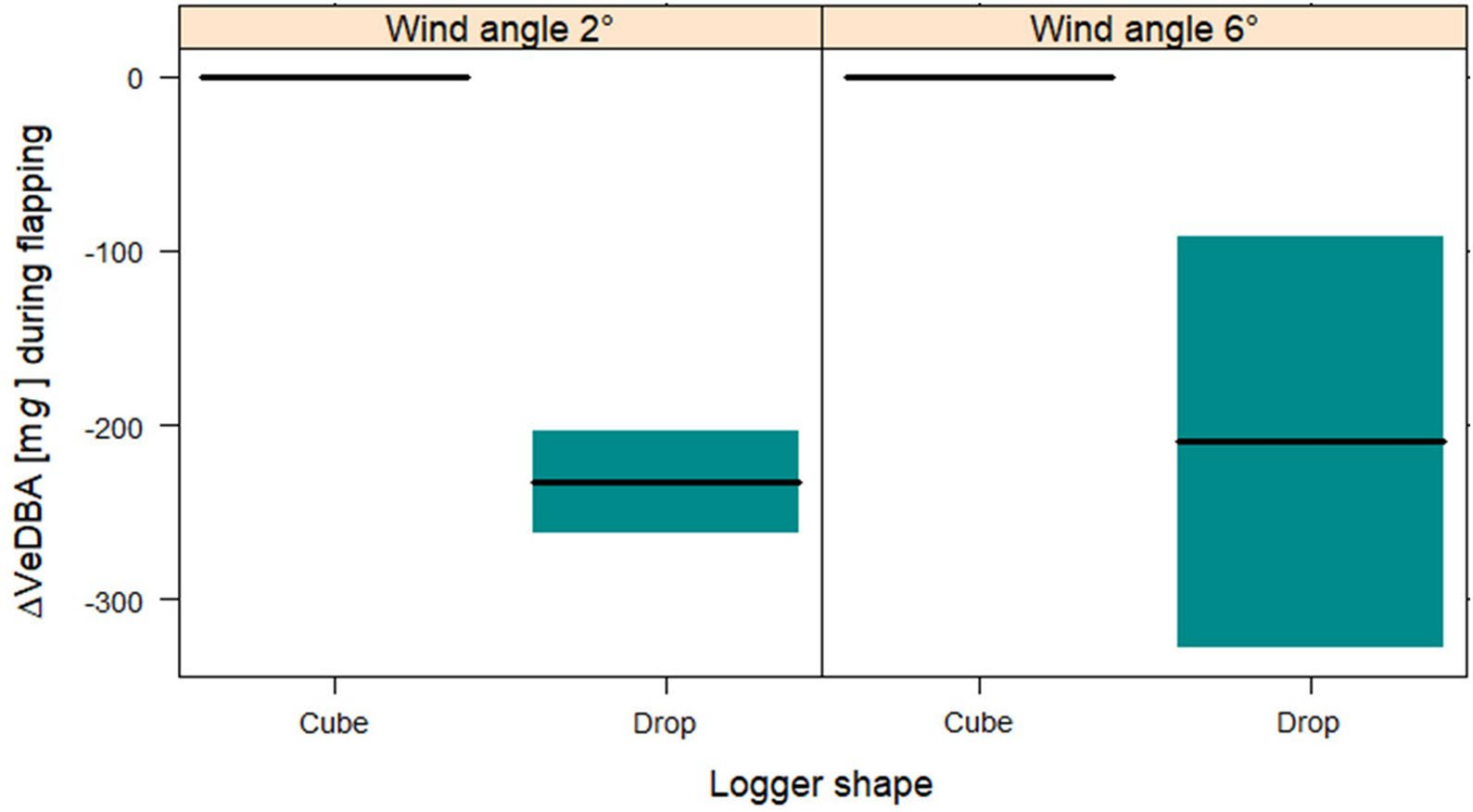
Contrast cross-sectional plots depicting the fixed effect of the GAMMs for Δ VeDBA (mg) during flapping, with an interaction between the categorical term ‘Wind angle’ and ‘Logger shape,’ on the horizontal axis. Wind angles + 2° = minimal updraft, Wind angle + 6° = slight updraft wind. Logger shape Cube = non-aerodynamic, Logger shape Drop = aerodynamic. Colored areas around the lines: 95% confidence intervals

During gliding the VeDBA values were strongly skewed toward lower values, whereas during flapping the distribution of VeDBA spread over a larger range of values. This could suggest different amplitudes of wing beat due to the different logger shapes and wind angles. We therefore fitted a GAMM to measure the effect of different logger shapes and wind angles on VeDBA only during flapping (Table 1). The aerodynamic logger had a clear positive effect on flapping performance (Table 5, Fig. 6). VeDBA was significantly lower during flapping (*t* = − 2.48, *p* < 0.05). Wind angle had a strong effect on VeDBA during flapping: values were lower at an angle of + 6° (*t* = − 5783, *p* < 0.001). Nonetheless, we found no shape-by-angle interaction (*t* = 0.393, *p* = 0.69). At both wind angles, the difference in VeDBA between the two logger shapes was identical.

**Table 5:** Results of a GAMM multiple regression model testing for the effects of different logger shapes (cube and drop) and flying at different wind angles (+ 2° and + 6°) on VeDBA during flapping

VeDBA during flapping			
Linear terms	Estimate	SE	*t*-value
Intercept	946.791	9.63	98.35***
Logger Drop	− 85.84	34.64	− 2.48*
Wind angle 6°	− 212.39	39.67	− 5.35***
Logger Drop:Wind angle 6°	28.12	58.77	0.39
Flight n. 2	28.99	5.01	5.78***

Smooth random terms		EDF	*F*-value
s(Bird)		0.03	0.19
s(Date): Logger Cube		5.09	14.71**
s(Date): Logger Drop		8.69	327.48**
s(Date):Wind2°		0.01	0.001
s(Date):Wind6°		4.64	385.77*
*R*^2^_adj_	0.2		
*n*	9350		
REML	64,602		

*edf* effective degrees of freedom of the smooth function terms, *fREML* restricted maximum likelihood; *p < 0.05; **p < 0.01; ***p < 0.001

### Field study during migration

#### Logger position and flight performance

**Fig. 7:**
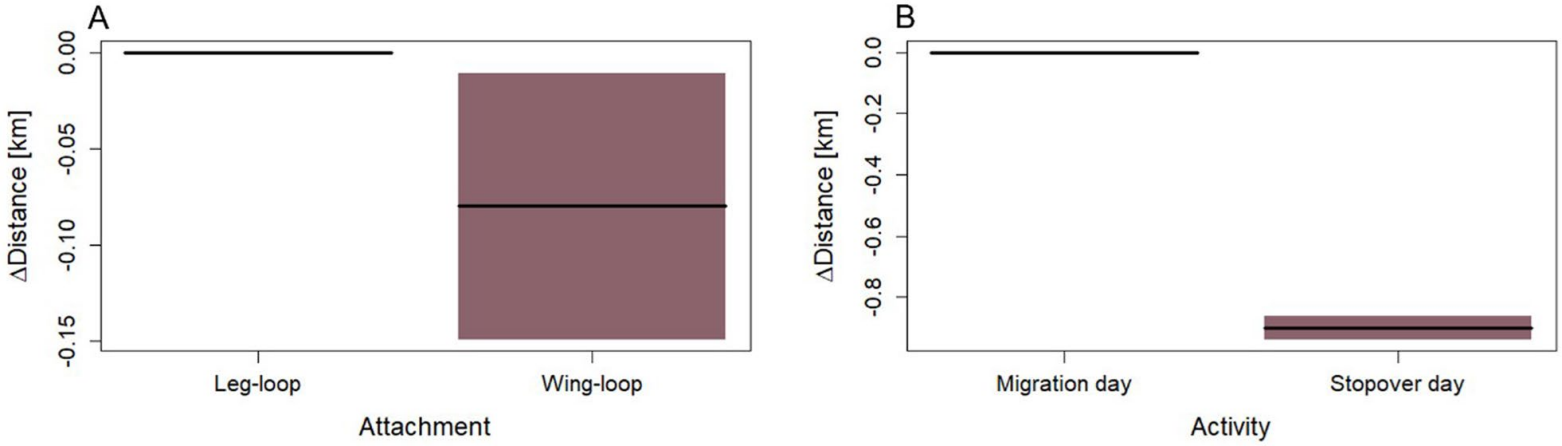
Contrast plots visualization Δlog Distance traveled of a regression function for GLM involving the fixed categorical explanatory variables of **(A)** Attachment position of harness and **(B)** Activity during migration period. Colored areas around the lines: 95% confidence intervals

We analyzed the spatio-temporal pattern of 37 free-flying Northern Bald Ibises during spring migration: 10 individuals carried their device with a wing-loop harness on the upper back; 27 individuals with a leg-loop harness on the lower back. The daily flight distance was significantly affected by the logger attachment position (*t* = 2.484, df = 2.601 × 10^1^, *p* < 0.05). Birds equipped with the leg-loop harness covered significantly longer migration distances per day (130 km on average) compared to birds equipped with the wing-loop harness (113 km on average) (Table 6, Fig. 7, and Additional file 1: Fig. S10). The average daily flight distance during stopover did not differ between birds wearing a leg-loop harness (17.7 km) and those wearing a wing-loop harness (16.6 km).

**Table 6:** Results for a Linear mixed model fit by REML

Fixed terms	Estimate	SE	*t*-value
Intercept	1.588	0.0156	101.522***
Attachment	0.039	0.0156	2.484*
Activity	0.449	0.0087	51.908***
Attachment × activity	0.001	0.0087	0.141

Random effect	Variance	SD
Bird	0.0038	0.004
Residual	0.0634	0.063

*t*-tests use Satterthwaite’s method. Attachment = leg-loop harness or wing-loop harness; Activity = stopover days and migratory days (*n* = 37 birds, number of observations = 1060) *p* < 0.1; *p < 0.05, **p < 0.01, ***p < 0.001

We found no interaction between logger position and activity (i.e., migration and stopover phases; *t* = 0.141, df = 1.053 × 10^3^, *p* = 0.89) (Additional file 1: Fig. S10).

## Discussion

We showed how different shapes of logging devices and their position on the animal can affect the flight performance and physiology of Northern Bald Ibises. In the wind tunnel study, the dimension of the two tag covers was equal although somewhat larger than usually used for studying Northern Bald Ibis [35]. Nevertheless, rounding the front and making the back of the cover pointed significantly helped reduced energy costs during flight. Our results from the wild population of Northern Bald Ibis provide compelling evidence of how small changes, such as changes in the logger position, can make a big impact on flight performances and thus on the birds' fitness.

### The impact of logger housing shape

Birds flying in a near horizontal airflow (+ 2°) with a drop-shaped device tended to have a lower heart rate—as a proxy for energy consumption [35]—compared to birds flying with a cube-shaped device. When flying in an updraft (+ 6°) the difference in the heart rate of flights with the two devices becomes significant (Fig. 3, Table 3).

This outcome corresponds with the general finding that aerodynamically or hydrodynamically shaped devices significantly reduce drag and thus minimize the impact of biologging devices. The mechanical power requirements for seabird flight were estimated to be 17–19% higher with non-streamlined devices and only 5% higher with streamlined devices compared to unequipped birds [50]. Computational fluid dynamics modeling with Grey Seals (*Halichoerus grypus*) as a model species [23] indicates that conventional tags can induce up to 22% more drag while swimming than streamlined tags.

In our study, we also measured VeDBA as a parameter based on body movement. It represents another proxy for energy expenditure, along with heart rate, and it enables accurately classifying active flapping flight and passive gliding [35]. We found that wind angle strongly affected VeDBA during flapping flight: the values were considerably lower when flying in an updraft (+ 6°) versus a horizontal airflow (+ 2°). At horizontal airflow, the results of the VeDBA with the two different covers differed slightly from the heart rate. This difference could be explained by the fact that not all energy expenditure is used for mechanical work during flight [35].

Furthermore, at both wind angles, VeDBA was significantly lower during flapping when the birds carried a drop-shaped device. Accordingly, the aerodynamic shape has a significant positive effect on the flapping performance and thus on the energy requirements during active flapping flight.

Several studies indicate that the attachment of devices on the back of a bird influences the overall aerodynamics during wing flapping flight [26]. However, the lack of empirical data makes interactions between the airflow over the wings and the airflow over the body during flapping flight—and the effect of differently shaped devices on this aerodynamics—an open field of research [10, 24].

Measuring overall dynamic body acceleration (VeDBA) enabled distinguishing active flapping flight from passive gliding in the wind tunnel (Additional file 1: Fig. S11). Importantly, changes in heart rate coincided with changes in the use of gliding. During flights with a drop-shaped device, the heart rate was lower, and the proportion of gliding was higher compared to flights with a cube-shaped device. The effect was much more pronounced in the + 6° updraft, which favors gliding. Accordingly, the heart rate was lowest and the gliding rate highest during flights with drop-shaped devices in the + 6° updraft. Under these conditions, the birds could glide up to 75% of the flight. In contrast, at a horizontal wind of + 2° the birds with the aerodynamic device glided only up to 30% (Fig. 4).

The impairment of gliding ability has implications particularly when biologging birds that perform thermal soaring or intermitted flap-gliding flight, where phases of active wing flapping are regularly interrupted by gliding phases [10, 35, 52]. These flight techniques are used by many bird species during migration, including the Northern Bald Ibis, which has been shown to use both techniques [53, 54]. The obvious functional context is the significant energetic advantage of gliding or soaring compared to active flapping flight. Gliding requires between 1.5 and 2 times the basal metabolic rate, compared to an estimated 8–30 times that rate for active flapping [47].

### The impact of logger attachment position

We found that wild Northern Bald Ibises of the European migratory release population [31, 55] that were carrying solar-powered biologging devices fixed by a leg-loop harness on the lower back, migrate significantly longer distances per day compared to conspecifics carrying the device on the upper back, fixed by a wing-loop harness. Considering that the migration route of Northern Bald Ibises is about 1000 km from the breeding site to the wintering site [31, 55], equipping the birds with a leg-loop harness will reduce the total migration duration by 15% compared to when a wing-loop harness is used.

This result corresponds with the outcome of wind tunnel studies with a Common Swift dummy (*Apus apus*) [10] and Great Cormorant (*Phalacrocorax carbo)* [56], where the drag was considerably higher when a device was attached on the upper back compared to a more caudal position. Northern Gannets (Sula bassana) which also perform an intermittent flight pattern much like Northern Bald Ibises showed major effects to the addition of tags at different positions [51]. Attaching small accelerometers and dummy logger devices caused more transitions between flapping and gliding phases and higher VeDBA values, particularly during gliding, while the devices were back mounted compared to tail mounted. Similarly, individuals of wild Cape vultures (*Gyps coprotheres*) equipped with patagial tags reduced traveled distances and speed compared to individuals equipped with leg band tags [57].

**Fig. 8:**
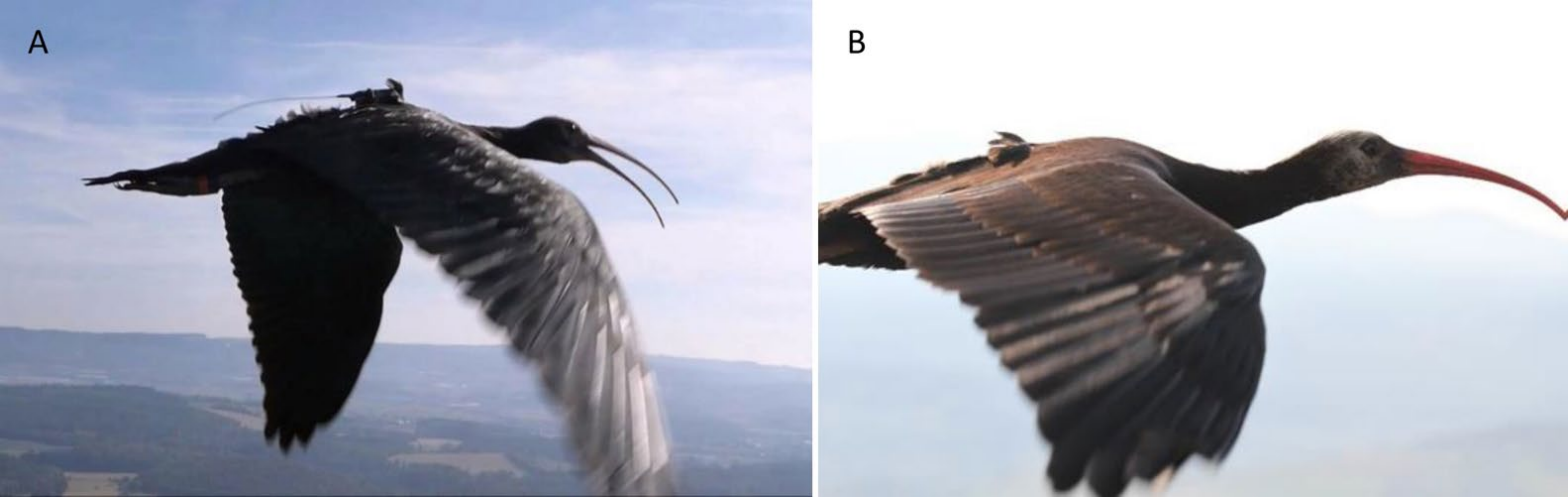
Migrating juvenile Northern Bald Ibis equipped with a biologging device **(A)** attached by a wing-loop harness on the upper back and **(B)** attached by a leg-loop harness on the lower back. At the upper-back position, feathers behind the device constantly flapped due to air turbulence caused by the separation of the boundary layer over the posterior upper part of the body. At the lower back position, the feathers lie more smoothly on the body and show no signs of major turbulence. Images courtesy of *Waldrappteam Conservation & Research* (www.waldrappteam.at)

No method is currently available to measure the device-induced drag directly in wild animals during flight. However, the human-led migration in the frame of the European LIFE reintroduction project [55] enables researchers to fly with the birds and observe them close up. These observations clearly revealed that devices attached to the upper back by a wing-loop harness caused a constant flapping of the feathers along the entire back below the device. Such disturbances were minimal when devices were attached more caudally by a wing-loop harnesses (J Fritz pers. com.; Fig. 8). Feather flapping is a clear sign of turbulence and separation of the boundary layer, which in turn points to an increased drag.

Solar transmitters in particular are preferably attached to the upper back to improve the sun exposure to the solar panels. Nevertheless, in addition to the obvious aerodynamic issues, this position also has the disadvantage of progressive opacity of the eyes’ cornea up to, and including, blindness [18].

## Conclusion

Our data demonstrate that the shape and position of biologging devices significantly influences the birds’ drag coefficient and thus their energy expenditure. Unfavorable shape and positioning goes beyond requiring more powerful wing flapping. The energetically probably more important effect is that the devices impair the birds’ ability to glide or soar, forcing them to more frequently perform the energetically much more demanding flapping flight.

Moreover, our study with the free-flying Northern Bald Ibises provides evidence that the attachment position on the birds’ back can also significantly influence their flight behavior during migration. This may cause delays. Even more relevant, however, is that many migratory birds depend on the efficient use of updrafts to cross barriers such as mountain ranges or to reach a flight altitude that enables crossing more extensive water bodies. Thus, the increased drag by biologging devices may impact their survival rate [58, 59].

From our point of view, the presented results do not fundamentally question the application of biologging technologies. On the contrary, they show that detrimental effects can be reduced with relatively little effort. In particular, this involves a strictly aerodynamic design of the housing and increased consideration of aerodynamics when attaching the device to the body. Accordingly, biologging devices should preferably be attached to birds via a leg loop to the lower back than via a wing loop on the upper back, even if this affects the efficiency of the solar panels. This conclusion is further supported by the above-mentioned study on Unilateral Eye Opacity [18].

The impact of biologging devices on the aerodynamics or hydrodynamics of animals is still poorly understood. This stands in marked contrast to the ever more extensive use of such technologies. More research is required here, in the wind tunnel, through simulations and, where possible, in the field. For example, visualizing the airflow around the bird’s body during flight in a wind tunnel and supplementary computational fluid dynamics modeling could bring significant progress on this issue. Comparative studies also call for publications to provide detailed information on the shape and type of attachment in addition to the weight of the devices.

### Supplementary Information

**Additional file 1.**

## Data Availability

Data for this study can be found in the PHAIDRA https://www.vetmeduni.ac.at/en/bibliothek/infoservice/phaidra/.
