# Supplementary material for: The impact of shape and attachment position of biologging devices in Northern Bald Ibises
Source: Anim Biotelemetry. 2023 Mar 9;11(1):8. doi: 10.1186/s40317-023-00322-5 (PMC11116193; doi:10.1186/s40317-023-00322-5)
Supplement: Supplementary file 1 — Additional file 1. [file 40317_2023_322_MOESM1_ESM.docx]

# **Additional file: Additional tables and figures**


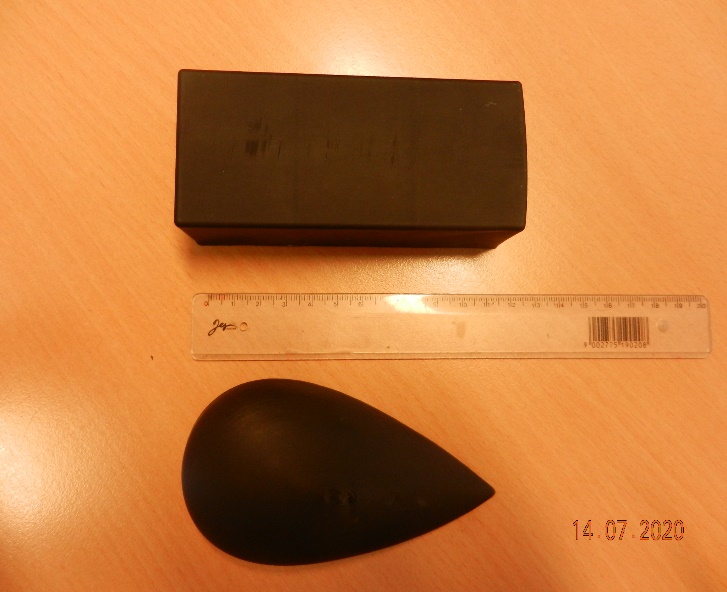


**A**


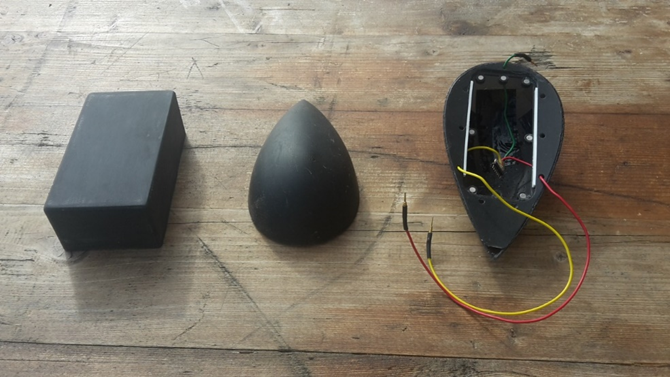


**B**

**
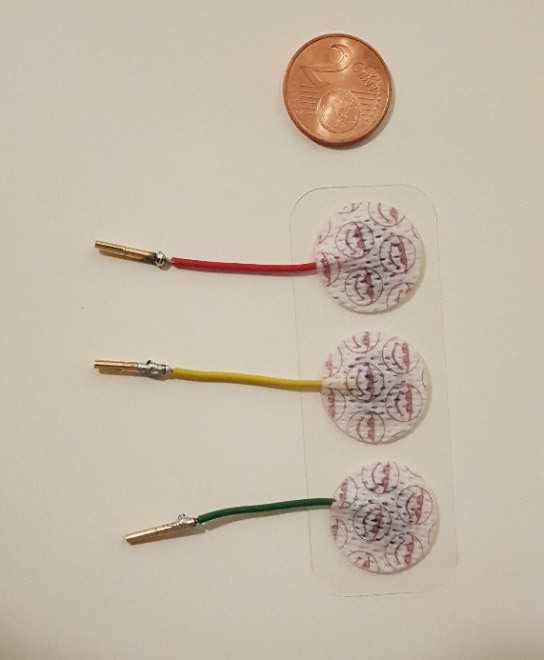
**

**C**

**
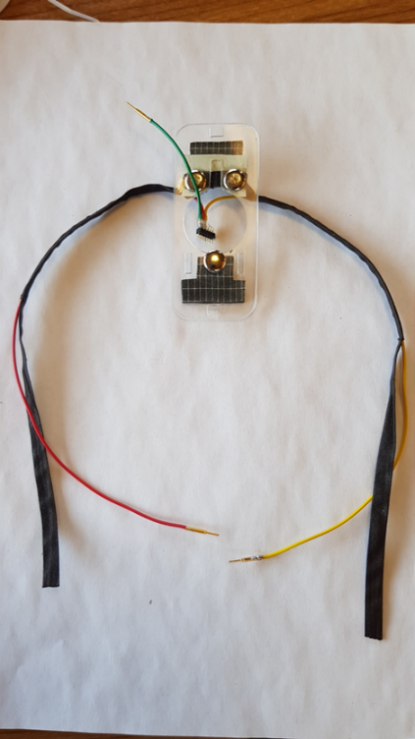
**

**D**

**
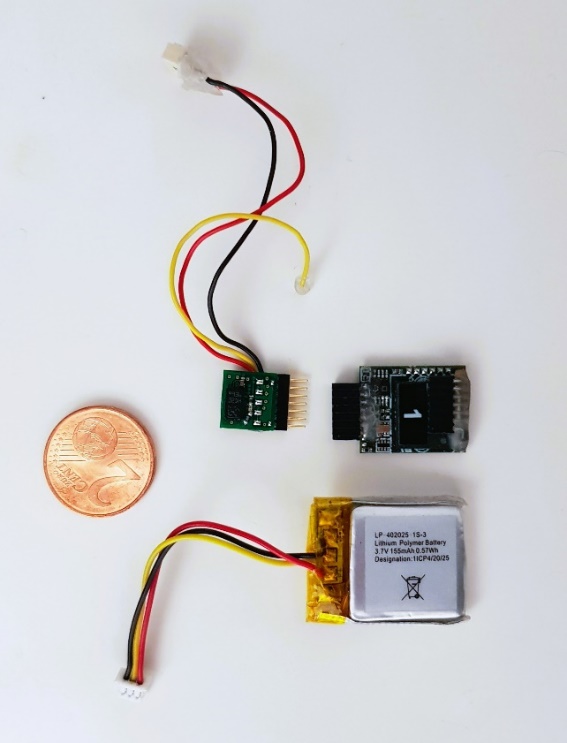
**

**E**

**Figure S1. (A-B)** View of the 3D printed covers; on the left is a cube-shape cover (non-aerodynamic), and on the middle and right are drop-shape (aerodynamic) covers from a different direction. The dimensions of both covers length=12 cm, width=5 cm, height=3 cm. The 3 wires that connect the electrodes and heart rate logger can be seen in the aerodynamic cover. **(C)** Three lead wires with adhesive mini electrodes (NeoLead®, ©Connect Medizintechnik). The adhesive mini electrodes were soldered to golden connectors that can be connected to the 3 wires shown in figure 1A. **(D)** Leg-loop harness with a plastic plate. The loggers and covers were fixed on the plastic plate. The electrode wires were fixed inside the Teflon tubes on the harness. **(E)** On the top left an external ECG logger Neurologger 2A with a 1 GB memory (Evolocus LLC) to be connected with the accelerometer unit; bottom left; and a lithium battery; right.

**Figure S2.** Histogram showing the bimodal distribution of distances flown by the forty-one ibises during migration. The visual dip at 1.5, means approximately 30km, separating the two underlying unimodal distributions of stationary and migratory days.

**Figure S3.** Histogram showing the distribution of distances flown by Northern Bald Ibises on **(A)** stationary days (Orange) and **(B)** migratory day (Turquoise).

A

B


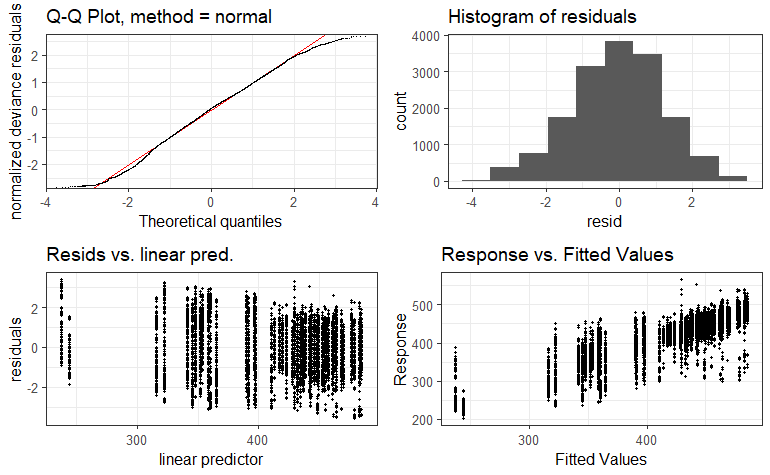


B

D

C

A

**Figure S4.** Model-checking plots for **GAMM heart rate** fitted to the heart rate during flights in relation to the different logger shapes and wind angle. The QQ plot and histogram of residuals confirm relatively normality **(A, B)**. The relationship between the residuals versus fitted values (linear predictor) is sufficiently flat and evenly distributed around zero to indicate that the variance is approximately constant as the mean increases **(C)**. Deviance residuals against fitted values show a positive linear relationship with a good deal of scatter **(D)**.


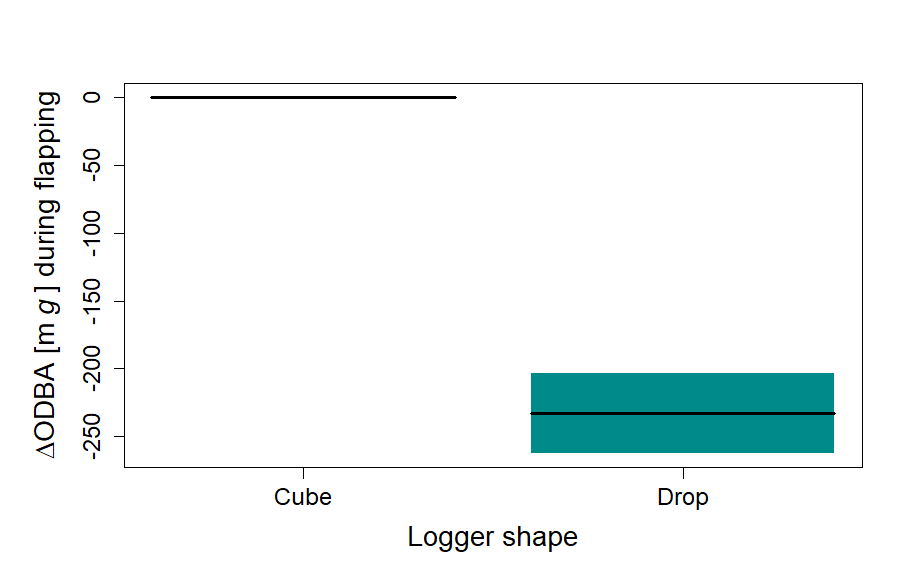

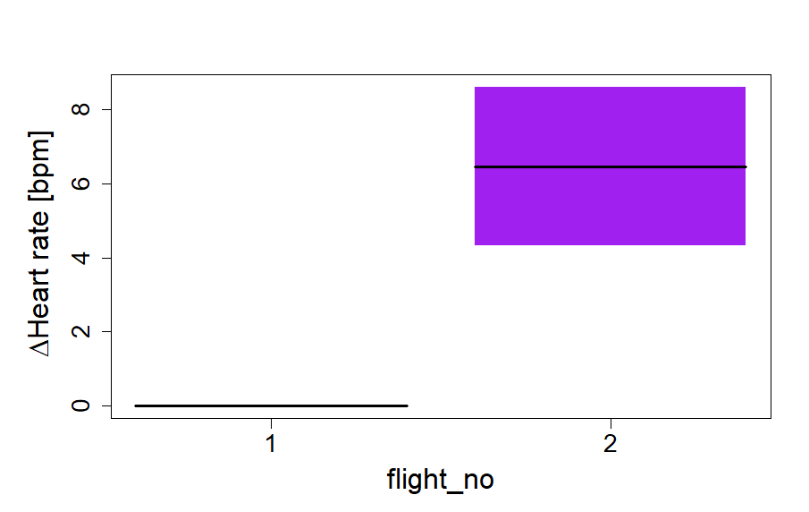

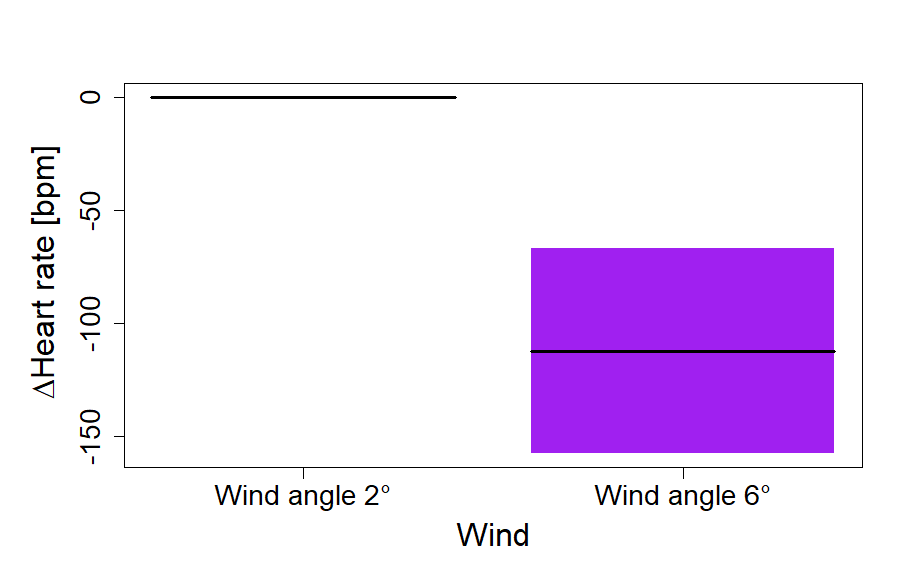

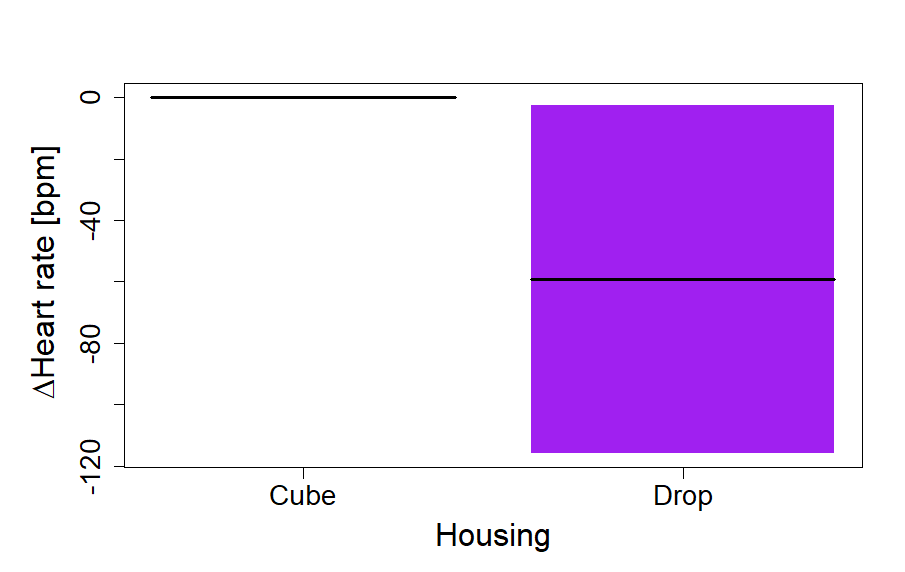


B

C

A

**Figure S5.** Contrast plots visualization Δ **Heart rate** of a regression function for **GAMM** involving fixed categorical explanatory variables of **(A)** logger shape **(B)** wind angle, and **(C)** flight number in a session. Wind angles 2°=minimal updraft, Wind angle 6°= a bit of updraft wind. Logger shape Cube= non-aerodynamic, Logger shape Drop= aerodynamic. Coloured areas around the lines indicate 95% confidence intervals.


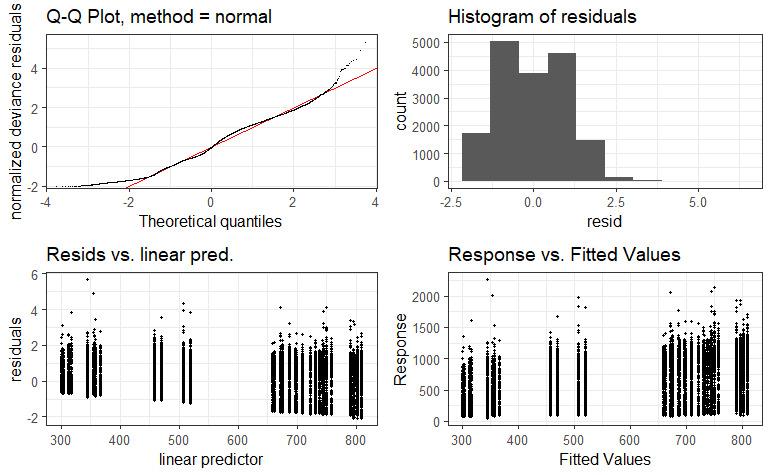


B

A

D

C

**Figure S6.** Model-checking plots for **GAMM VeDBA** fitted to the heart rate during flight in relation to the different logger shapes and wind angle. The QQ plot and histogram of residuals confirm relatively normality **(A, B)**. The relationship between the residuals versus fitted values (linear predictor) is sufficiently flat and evenly distributed around zero to indicate that the variance is approximately constant as the mean increases **(C)**. Deviance residuals against fitted values show a positive linear relationship with a good deal of scatter **(D)**.


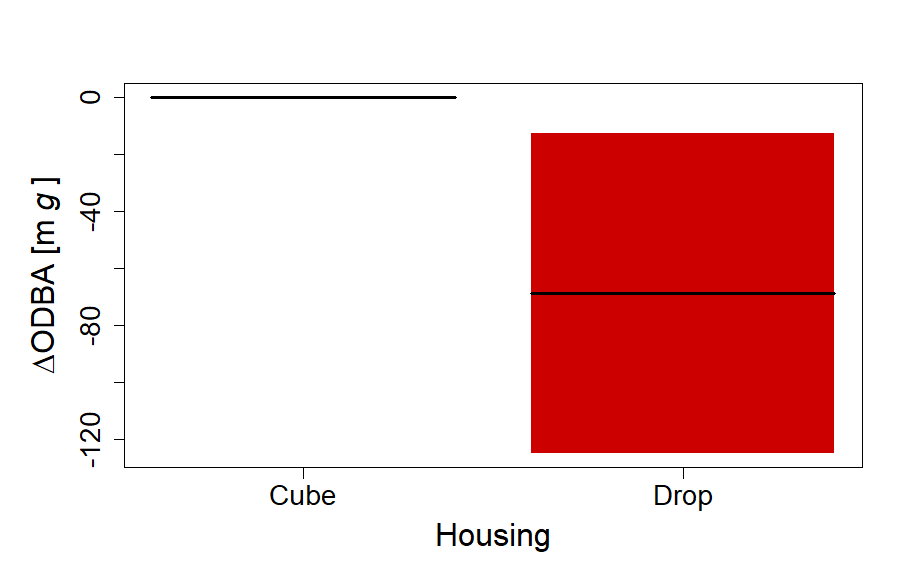

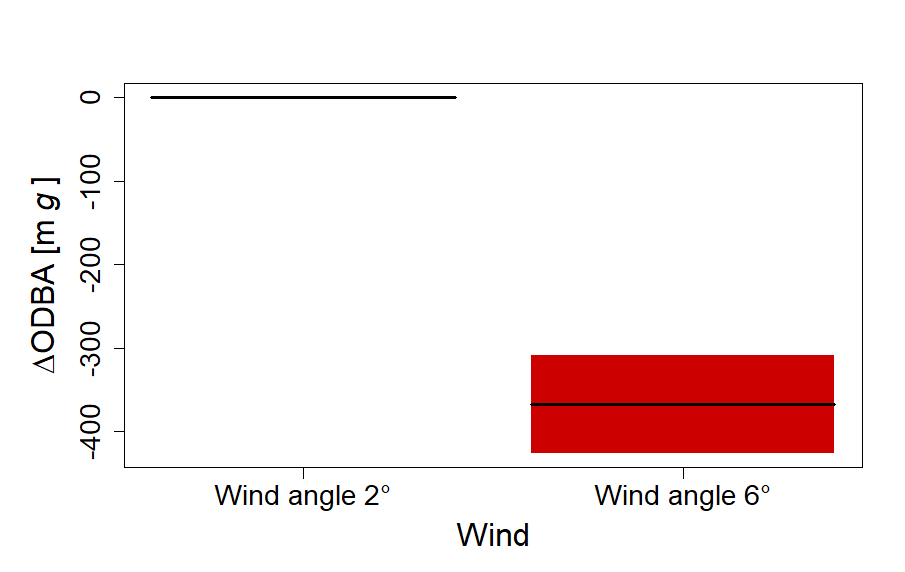

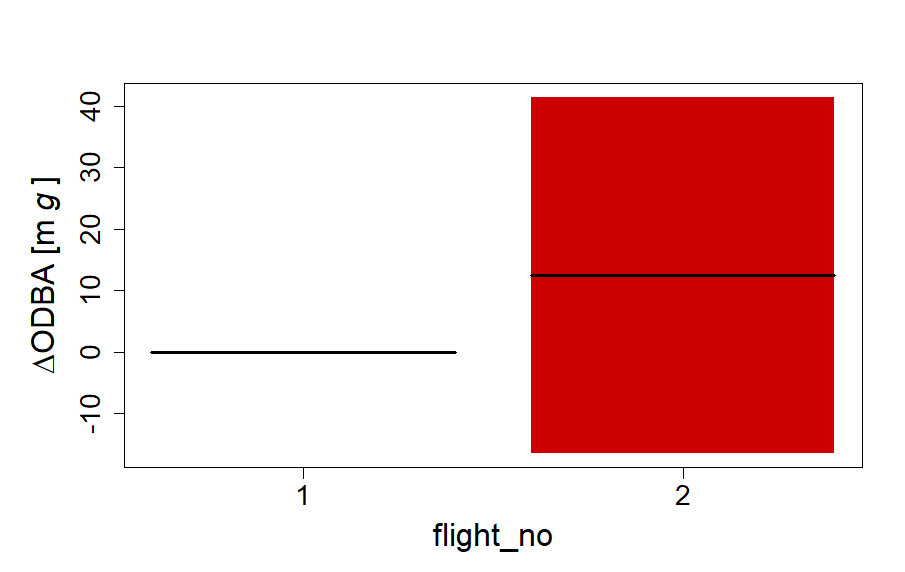

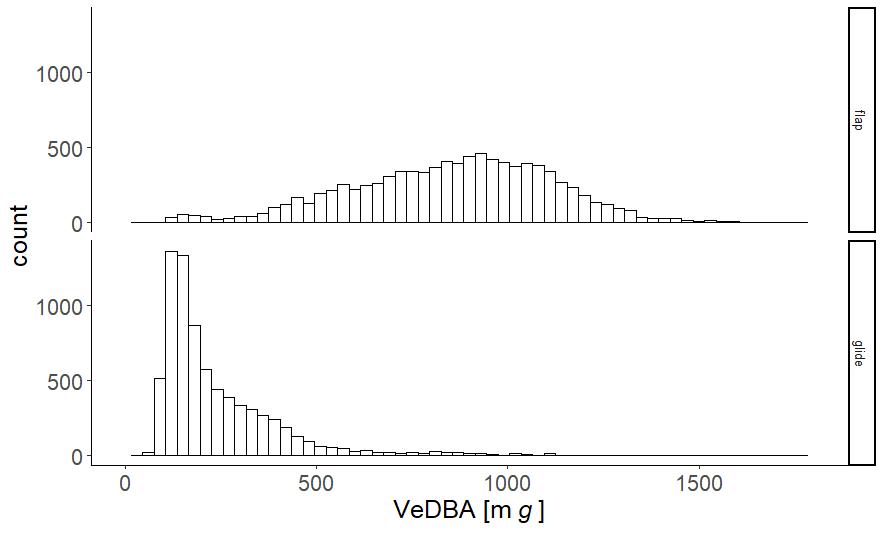

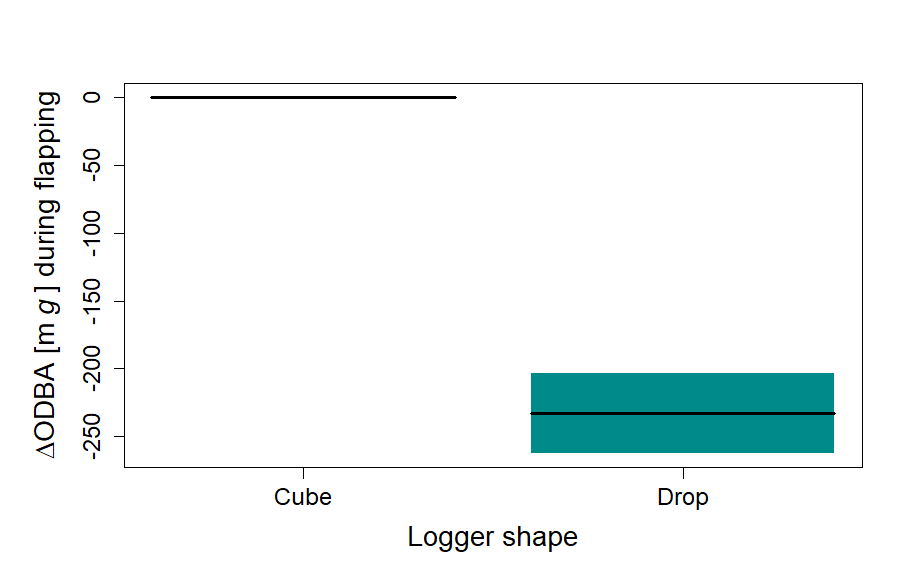

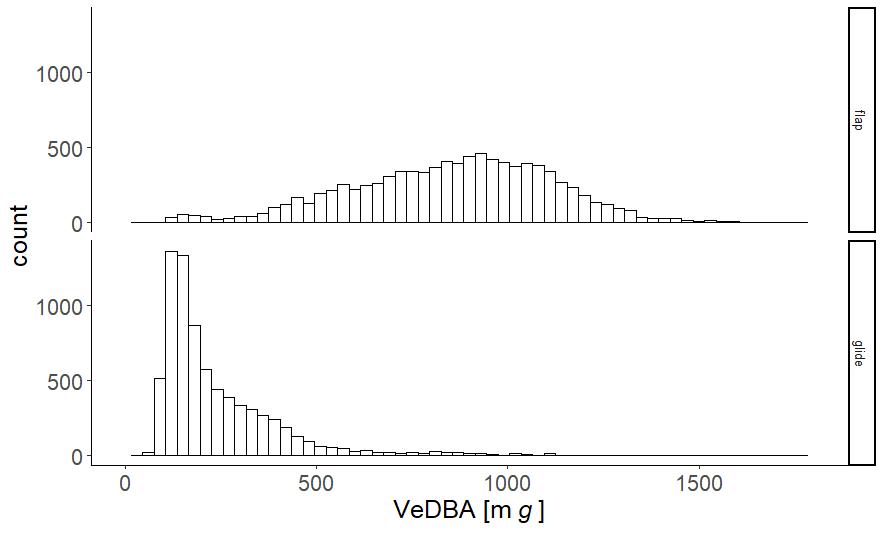

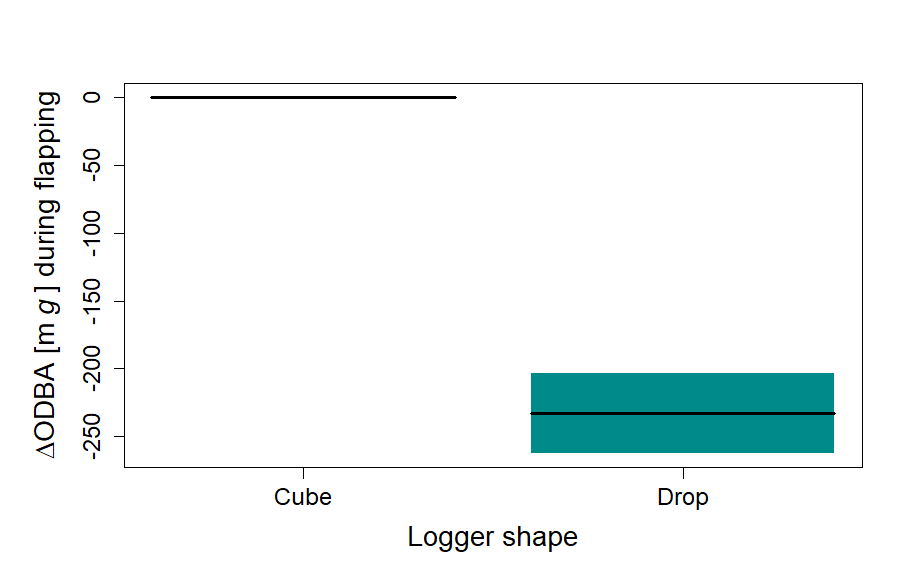

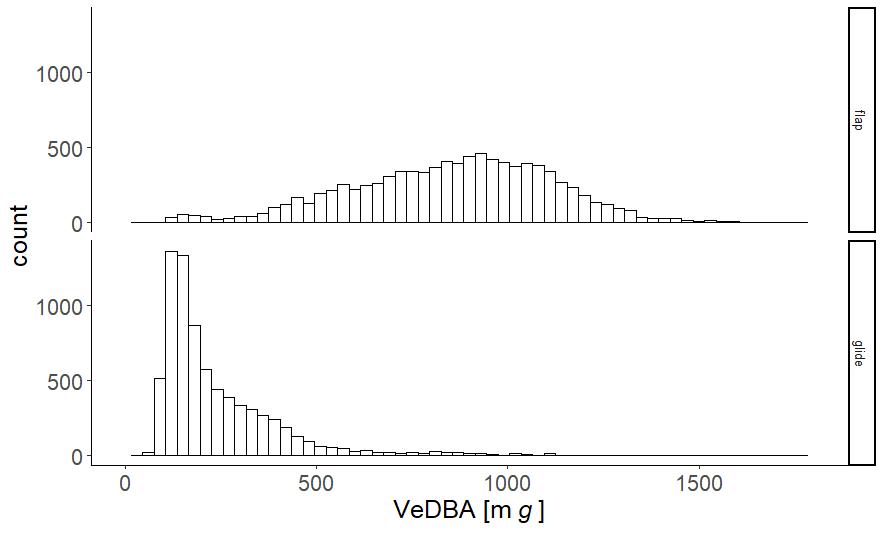

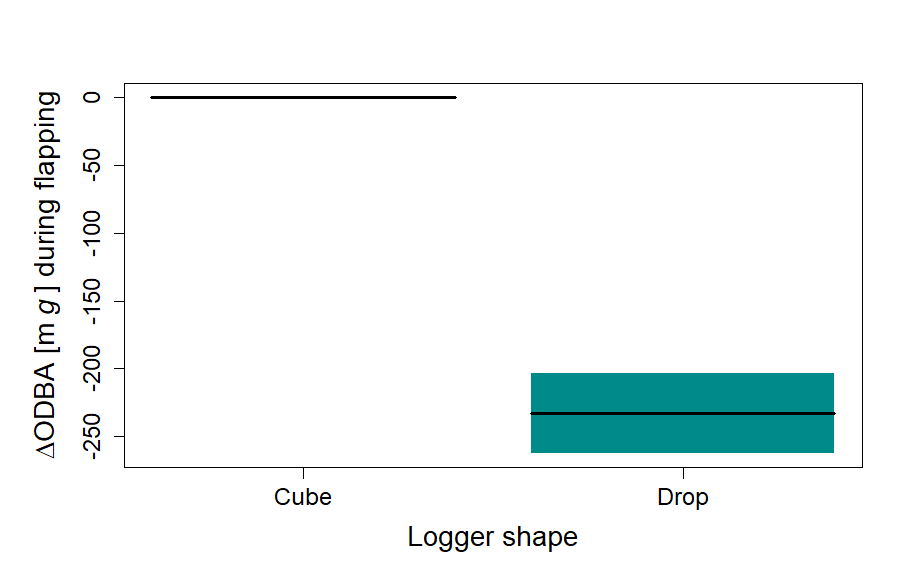


A

B

C


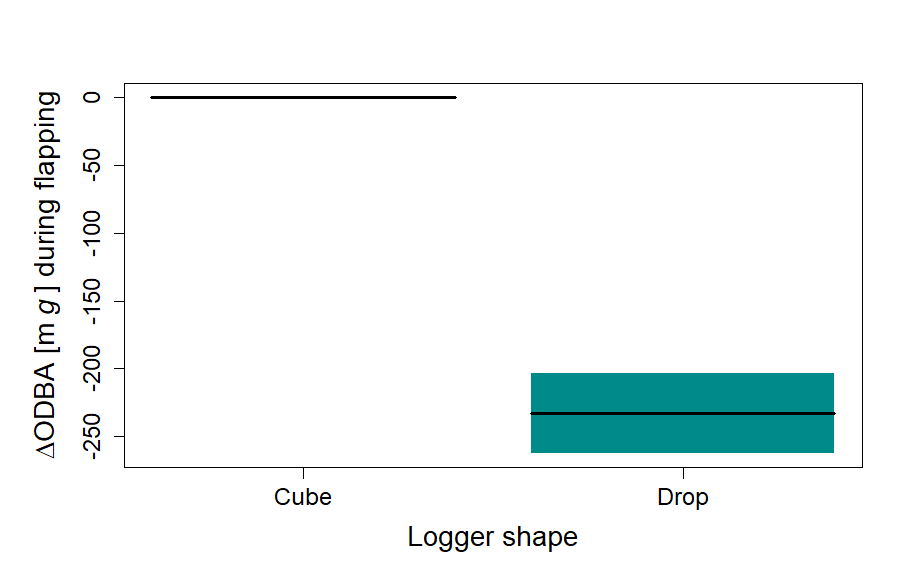


**Figure S7.** Contrast plots visualization Δ **VeDBA** of a regression function for **GAMM** involving the fixed categorical explanatory variables of **(A)** logger shape **(B)** wind angle, and **(C)** flight number in a session. Wind angles 2°=minimal updraft, Wind angle 6°= a bit of updraft wind. Logger shape Cube= non-aerodynamic, Logger shape Drop= aerodynamic. Coloured areas around the lines indicate 95% confidence intervals.


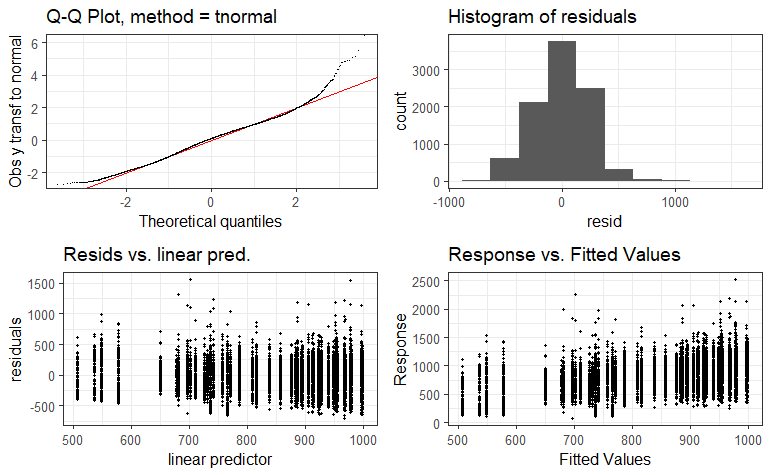


C

D

B

A

**Figure S8.** Model-checking plots for GAMM **VeDBA during flapping** fitted to the heart rate during flight in relation to the different logger shapes and wind angle. The QQ plot and histogram of residuals confirm relatively normality **(A, B)**. The relationship between the residuals versus fitted values (linear predictor) is sufficiently flat and evenly distributed around zero to indicate that the variance is approximately constant as the mean increases **(C)**. Deviance residuals against fitted values show a positive linear relationship with a good deal of scatter **(D)**.


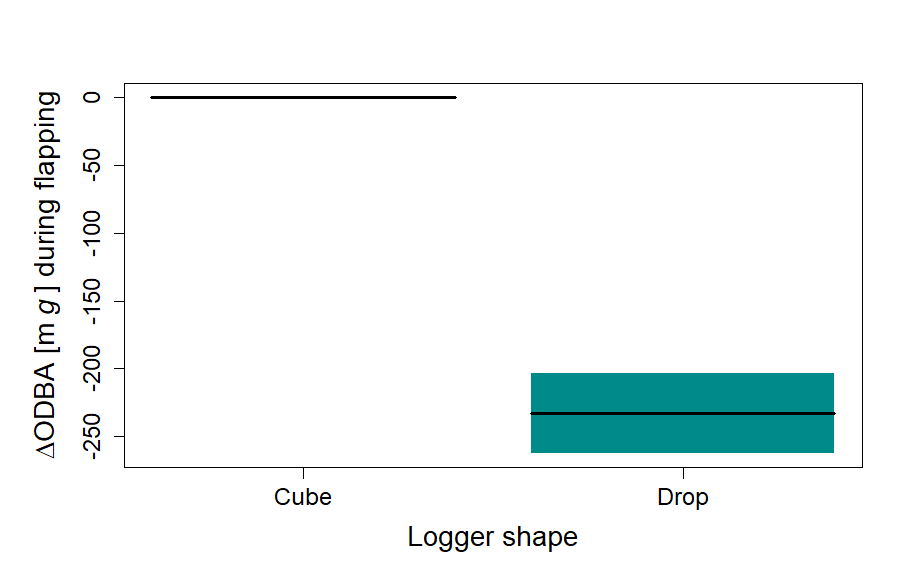

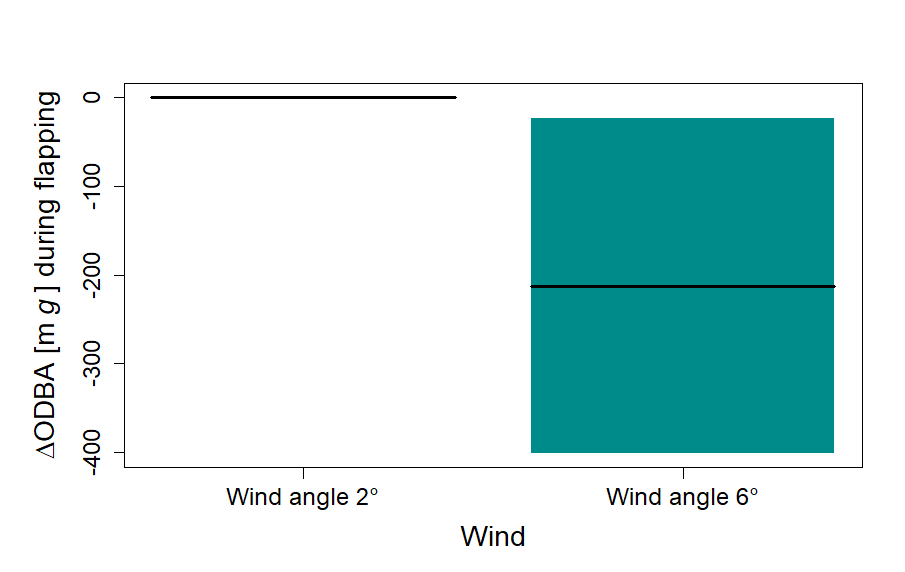

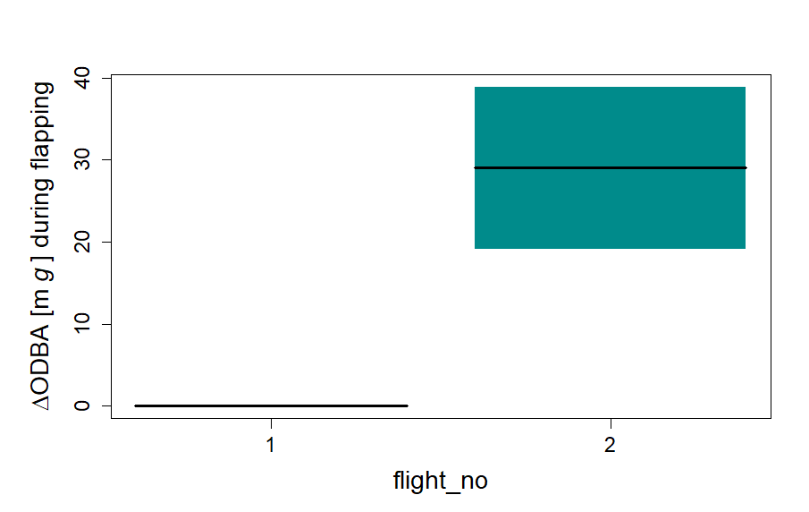

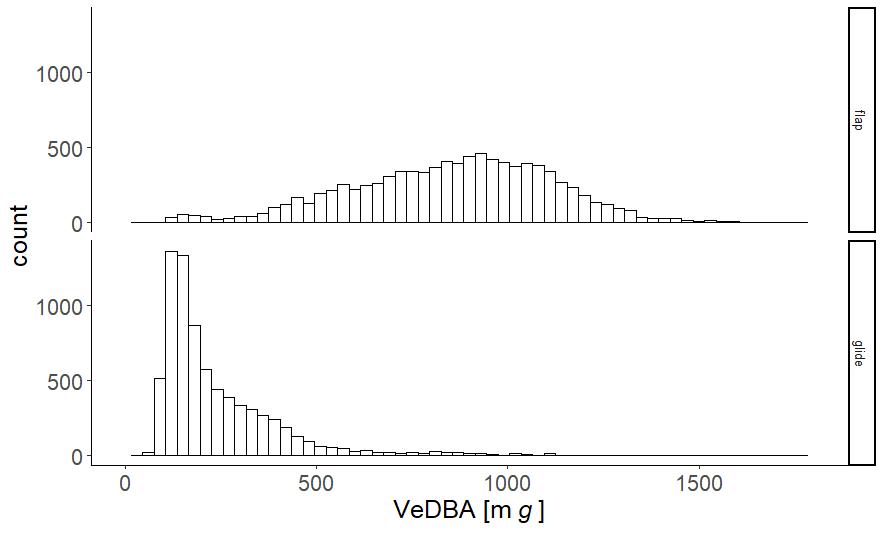

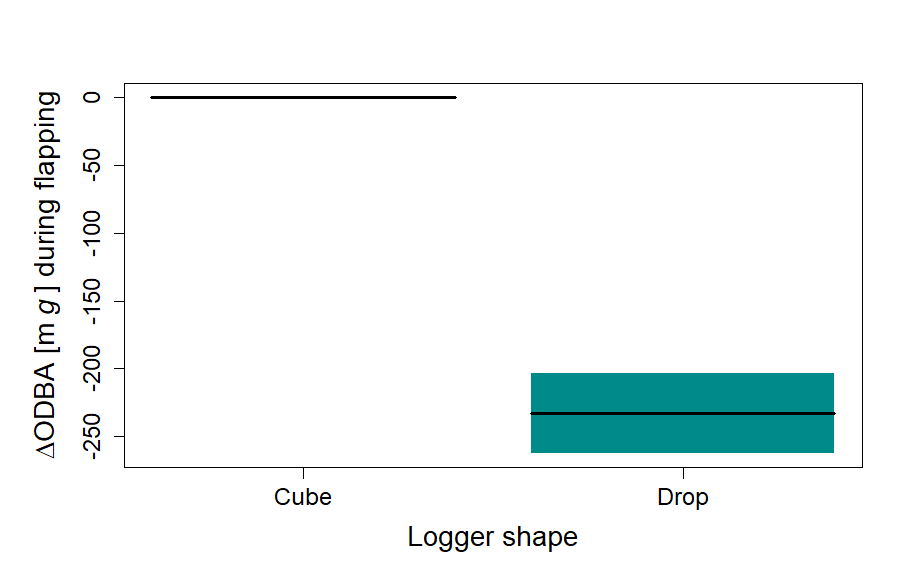

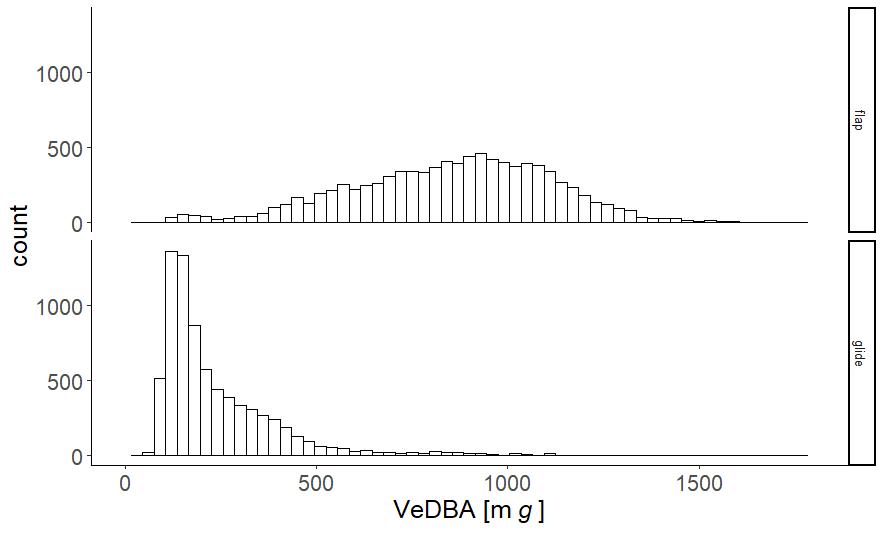

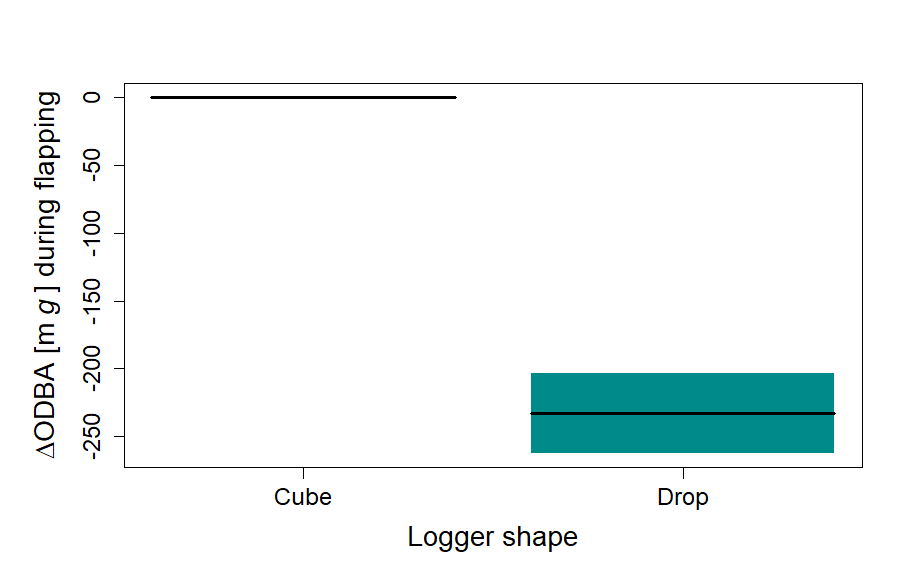

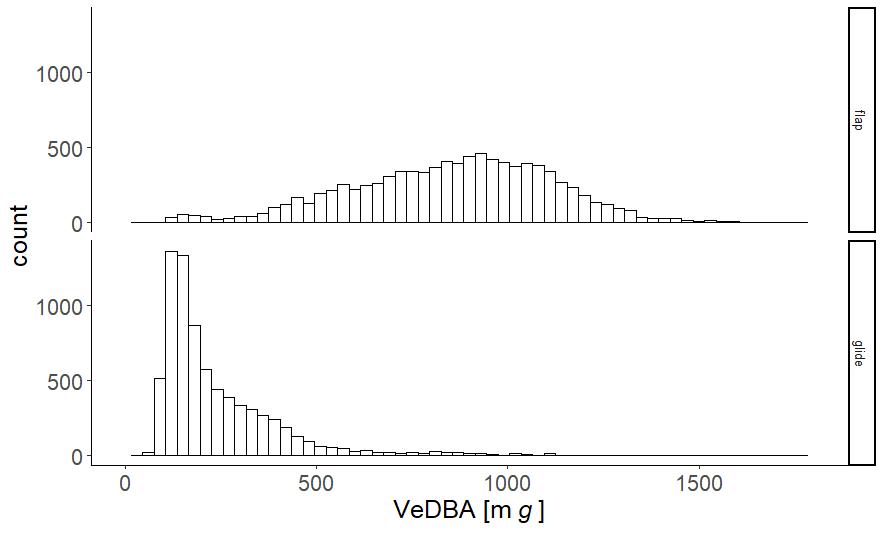

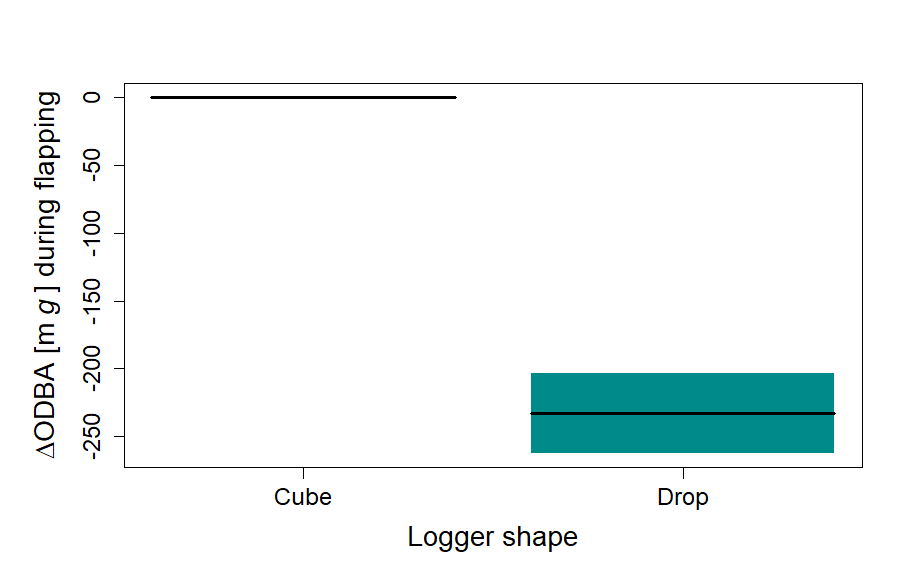


B

A

C

**Figure S9.** Contrast plots visualization Δ **VeDBA during flapping** of a regression function for **GAMM** involving the fixed categorical explanatory variables of **(A)** logger shape **(B)** wind angle, and **(C)** flight number in a session. Wind angles 2°=minimal updraft, Wind angle 6°= a bit of updraft wind. Logger shape Cube= non-aerodynamic, Logger shape Drop= aerodynamic. Coloured areas around the lines indicate 95% confidence intervals.


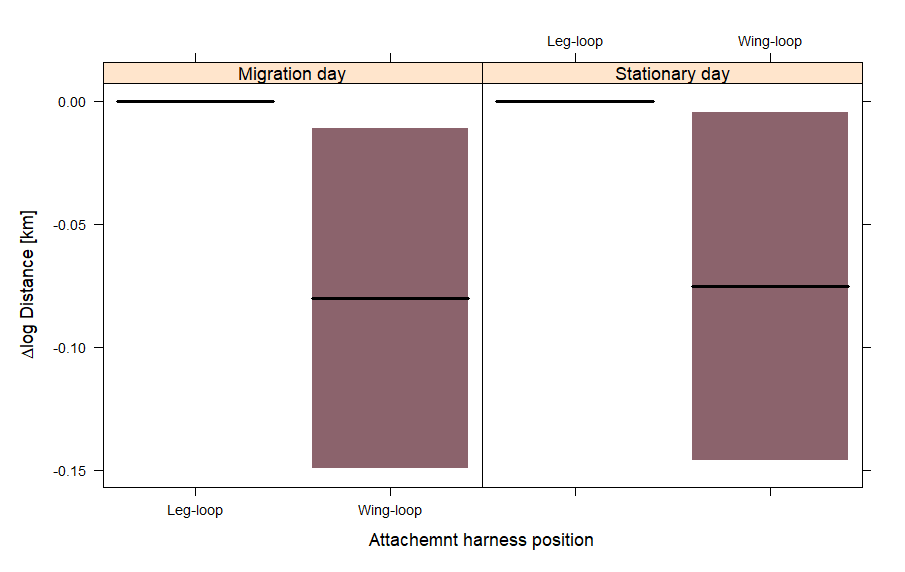


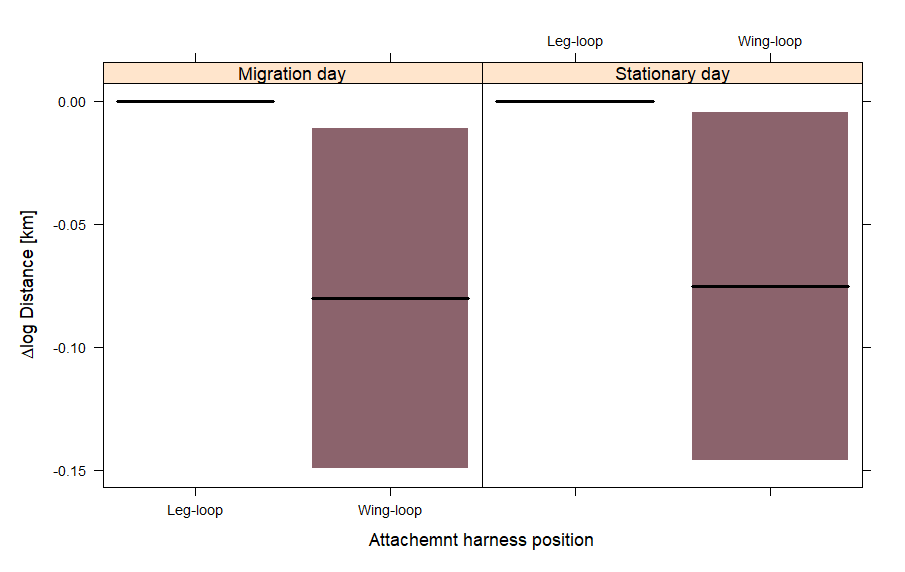


**Figure S10.** Contrast cross-sectional plots depicting the fixed effect of the GLM for Δ log distance (km) during migration, with an interaction between the categorical term ‘Activity’ (migration day and stopover day), and ‘Attachment’ (leg-loop harness and wing-loop harness), on the horizontal axis. Coloured areas around the lines indicate 95% confidence intervals.


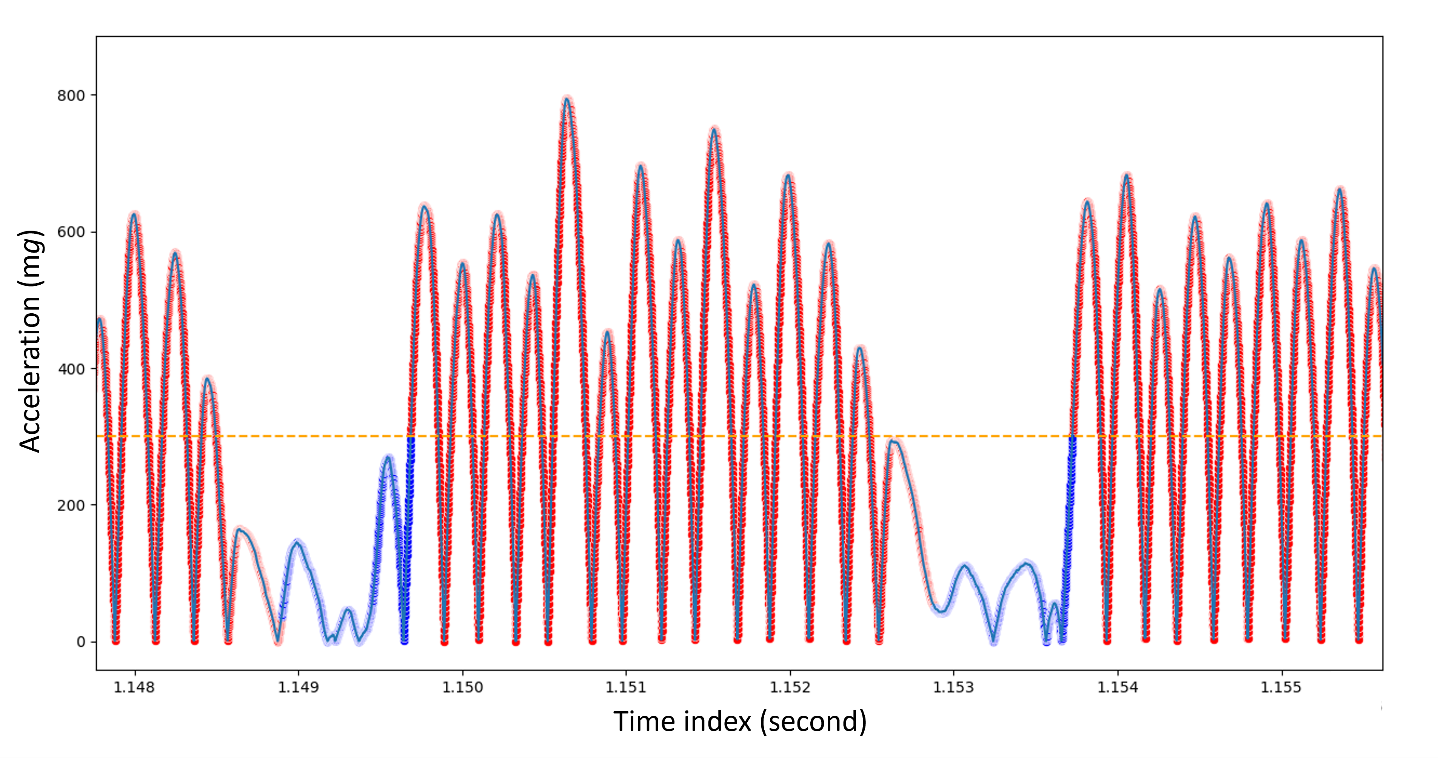


**Figure S11.** Example of a flight performed by a Northern Bald Ibis. Raw acceleration of the heave axis (absolute value). The red points denote flapping blue points gliding. The dashed yellow horizontal line represents the threshold of 300mg to define flapping and gliding.

**Supplementary Table 1.** example from one bird to calculate distance travelled. Distance: Daily flight path (km); Activity: Categorization of days in migratory (M) and stationary (S) days; Attachment: Wing-loop (W) or leg-loop (L) harness attachment; Bird: Identity of the ibis; Year: Year of migration; Day: Day of the year the distance was flown.

| **Distance (km)** | **Activity** | **Attachment** | **Bird** | **Year** | **Day** |
| --- | --- | --- | --- | --- | --- |
| 116.24 | M | W | Charlie | 2018 | 121 |
| 75.623 | M | W | Charlie | 2018 | 122 |
| 87.598 | M | W | Charlie | 2018 | 123 |
| 7.776 | S | W | Charlie | 2018 | 124 |
| 18.061 | S | W | Charlie | 2018 | 125 |
| 101.923 | M | W | Charlie | 2018 | 126 |
| 10.244 | S | W | Charlie | 2018 | 127 |

**Supplementary Table 2.** Data calculated with QGIS of the daily flight distance (km), for the wing-loop and leg-loop attachment groups. Bird: Identity of the ibis; Harness position: Wing-loop (W) or leg-loop (L) harness attachment; Activity: Categorization of migratory and stationary days; Total: Total days the migration lasted; Min M: Minimal distance flown on migratory days (km); Max M: Maximal distance flown on migratory days (km); Mean M: Mean of distances flown on migratory days (km); Min S: Minimal distance flown on stationary days (km); Max S: Maximal distance flown on stationary days (km); Mean S: Mean distances flown on stationary days (km).

| **Bird** | **Harness position** | **Migratory day (n)** | **Stationary day (n)** | **Total distance (n)** | **Min M (km)** | **Max M (km)** | **Mean M (km)** | **Min S (km)** | **Max S (km)** | **Mean S (km)** |
| --- | --- | --- | --- | --- | --- | --- | --- | --- | --- | --- |
| Charlie | W | 31 | 59 | 90 | 52.717 | 226.885 | 108.108 | 3.444 | 49.038 | 15.435 |
| Frieda | W | 27 | 14 | 41 | 52.852 | 281.362 | 105.257 | 9.788 | 46.448 | 27.219 |
| Jazu | W | 13 | 15 | 28 | 55.177 | 237.602 | 107.144 | 1.461 | 40.279 | 6.637 |
| Kuzko | W | 7 | 1 | 8 | 78.26 | 226.787 | 155.377 | 6.734 | 6.734 | 6.734 |
| Luna | W | 8 | 13 | 21 | 50.536 | 188.749 | 123.041 | 3.33 | 40.689 | 13.723 |
| Mika | W | 30 | 40 | 70 | 50.584 | 282.349 | 104.075 | 6.389 | 49.449 | 19.418 |
| Theo | W | 25 | 91 | 116 | 50.658 | 297 | 123.72 | 7.021 | 46.122 | 20.552 |
| Zoppo | W | 13 | 24 | 37 | 51.37 | 241.272 | 120.114 | 7.268 | 41.879 | 14.358 |
| *Camillo* | L | 14 | 11 | 3 | 53.366 | 350.635 | 96.127 | 23.71 | 43.666 | 31.101 |
| *Cassandra* | L | 17 | 10 | 7 | 53.207 | 350.793 | 74.878 | 7.464 | 49.514 | 27.49 |
| *Dante* | L | 8 | 7 | 1 | 73.745 | 249.312 | 115.322 | 20.951 | 20.951 | 20.951 |
| *Donatello* | L | 36 | 20 | 16 | 54.256 | 315.835 | 129.461 | 4.122 | 45.108 | 19.924 |
| *Fiona* | L | 10 | 10 | 0 | 55.257 | 184.817 | 138.916 | 0 | 0 | 0 |
| *Francesco* | L | 10 | 8 | 2 | 74.374 | 291.842 | 149.352 | 15.912 | 22.372 | 19.142 |
| *Greta* | L | 32 | 22 | 10 | 52.124 | 236.896 | 116.223 | 7.272 | 47.678 | 16.968 |
| *Jojo* | L | 8 | 8 | 0 | 73.715 | 249.697 | 115.161 | 0 | 0 | 0 |
| *Juno* | L | 18 | 8 | 10 | 66.844 | 208.983 | 121.604 | 3.297 | 42.781 | 21.055 |
| *Karl* | L | 56 | 14 | 42 | 52.345 | 216.541 | 103.09 | 2.41 | 41.491 | 19.418 |
| *Lancillotto* | L | 21 | 15 | 6 | 74.52 | 248.653 | 128.656 | 9.026 | 46.721 | 29.855 |
| *Leonardo* | L | 8 | 6 | 2 | 87.862 | 275.566 | 113.381 | 7.039 | 39.069 | 23.054 |
| *Leopold* | L | 41 | 21 | 20 | 52.848 | 212.011 | 118.062 | 4.227 | 48.385 | 22.916 |
| *Liethe* | L | 20 | 13 | 7 | 56.796 | 421.883 | 134.757 | 10.299 | 43.019 | 28.083 |
| *Lucius* | L | 8 | 7 | 1 | 68.602 | 294.328 | 124.255 | 11.575 | 11.575 | 11.575 |
| *Luigi* | L | 39 | 19 | 20 | 59.086 | 206.728 | 119.868 | 3.686 | 41.684 | 16.495 |
| *Lyra* | L | 53 | 30 | 23 | 51.465 | 211.982 | 121.935 | 5.025 | 42.998 | 17.144 |
| *Marvin* | L | 4 | 4 | 0 | 86.639 | 350.787 | 88.484 | 0 | 0 | 0 |
| *Nepomuk* | L | 4 | 4 | 0 | 85.167 | 188.36 | 116.11 | 0 | 0 | 0 |
| *Nova* | L | 5 | 5 | 0 | 150.85 | 298.073 | 236.94 | 0 | 0 | 0 |
| *Peter* | L | 7 | 5 | 2 | 78.584 | 307.509 | 134.146 | 10.272 | 16.562 | 13.417 |
| *Pino* | L | 34 | 25 | 9 | 52.447 | 294.808 | 134.739 | 11.58 | 47.755 | 23.733 |
| *Poncho* | L | 70 | 21 | 49 | 51.804 | 249.573 | 89.212 | 4.653 | 46.599 | 14.845 |
| *Salem* | L | 29 | 20 | 9 | 56.184 | 306.817 | 123.565 | 4.33 | 44.767 | 18.188 |
| *Salvatore* | L | 26 | 7 | 19 | 58.892 | 226.247 | 89.482 | 8.95 | 43.146 | 16.784 |
| *Sonic* | L | 51 | 13 | 38 | 62.855 | 278.311 | 132.044 | 4.142 | 42.694 | 13.545 |
| *Urmel* | L | 41 | 20 | 21 | 68.17 | 245.29 | 166.213 | 4.17 | 43.341 | 11.585 |
| *Vitorio* | L | 8 | 8 | 0 | 57.256 | 307.49 | 105.276 | 0 | 0 | 0 |
| *Wookiee* | L | 49 | 23 | 26 | 53.479 | 226.611 | 97.571 | 2.943 | 43.552 | 15.259 |
